# Intramedullary nail fixation versus open reduction and internal fixation for treatment of adult diaphyseal forearm fractures: a systematic review and meta-analysis

**DOI:** 10.1186/s13018-024-05158-0

**Published:** 2024-11-04

**Authors:** McKenna W. Box, Samuel D. Stegelmann, Grayson A. Domingue, Monica E. Wells, Neil J. Werthmann, Cornelis J. Potgieter, John T. Riehl

**Affiliations:** 1 Department of Orthopaedic Surgery, Medical City Denton, Denton, TX USA; 2 Department of Orthopaedic Surgery, University of Oklahoma Health Sciences Center, Oklahoma City, OK USA; 3 Department of Orthopaedic Surgery, University of Missouri-Kansas City, Kansas City, MO USA; 4 Texas Christian University Anne Burnett Marion School of Medicine, Fort Worth, TX USA; 5 Texas Bone and Joint, Fort Worth, TX USA

**Keywords:** Both-bone fracture, Intramedullary fracture fixation, Radius fracture, Ulna fracture

## Abstract

**Background:**

Diaphyseal radius and ulna fractures require surgical fixation in adults. Open reduction and internal fixation (ORIF) have been considered the gold standard of treatment. The recent development of an interlocking intramedullary nail (IMN) has provided an alternative treatment method for these fractures. The objective of this meta-analysis is to compare the outcomes and complications of IMN versus ORIF for diaphyseal forearm fractures in adults.

**Methods:**

MEDLINE and Embase were searched from January 1, 2000, through January 7, 2024. All English-language studies were included comparing radiographic and functional outcomes for interlocking IMN fixation and ORIF of diaphyseal forearm fractures in adults (age ≥ 18 years). Study demographics, fracture data, functional outcomes, radiographic outcomes, and complications were extracted. Study quality was determined using the ROBINS-I criteria for cohort studies and the Cochrane risk of bias 2.0 (RoB 2) tool for randomized controlled trials. Meta-analysis of included studies used odds ratios and standardized mean difference when appropriate. Data was analyzed using subgroups of all diaphyseal fractures (including isolated radius or ulna fractures) and those with BBFFs.

**Results:**

Nine studies were included for analysis. There were 42 isolated radius, 80 isolated ulna, and 116 both-bone fractures (BBFF) treated with IMN and 36 radius, 81 ulna, and 116 both-bone fractures treated with ORIF. Compared to ORIF, IMN of diaphyseal forearm fractures appeared to be associated with shorter operative times and a lower overall complication rate. Time-to-union and the rate of nonunion following IMN were similar to ORIF. According to the Grace–Eversmann score, functional outcomes tended to be better following IMN, but DASH scores were similar between fixation strategies.

**Conclusions:**

Our findings suggest that interlocking IMN can be a safe and effective treatment option for simple and complex diaphyseal forearm fractures in adults. Further high-quality studies are needed to define indications for treating diaphyseal fractures with an interlocking IMN.

**Level of Evidence:**

Therapeutic Level IV.

**Supplementary Information:**

The online version contains supplementary material available at 10.1186/s13018-024-05158-0.

## Introduction

Diaphyseal radius and ulna fractures require surgical fixation in adults [1–5]. Restoring alignment to < 10 degrees of angulation is crucial for adequate recovery and patient function [3]. The standard of care is open reduction and internal fixation with plates and screws (ORIF), which maintains axial and rotational alignment but requires extensive exposure and disruption of the soft tissues and periosteum. Common complications include nonunion, pain, and hardware irritation which may necessitate hardware removal and increase the risk of refracture [6–11].

Intramedullary nail (IMN) fixation is an alternative treatment option for diaphyseal forearm fractures, which has minimal soft tissue and periosteum disruption, smaller scars, fewer hardware-related complications, and minimal risk of refracture after removal [4]. Historical attempts at non-locking intramedullary fixation in adults did not provide rotational and length stability, leading to high nonunion rates [1, 9, 12]. Newer nail designs that utilize interlocking screws are meant to avoid these concerns. Current commercially available interlocking IMNs include the Foresight® nail (Smith and Nephew, Memphis, TN, USA), Acumed nails (Acumed, Hillsboro, OR, USA), and TST Rakor nails (TST Rakor, Istanbul, Turkey) [12–14].

When considering treatment options for diaphyseal forearm fractures, it is crucial to analyze IMN efficacy to ORIF. Lari et al. [15] recently performed a meta-analysis, but included the Talwalkar square nail, which does not have any interlocking screw, and hybrid fixation. To the authors’ knowledge, no systematic review or meta-analysis has compared the results of IMN with interlocking screws and ORIF in adults with diaphyseal fractures. This review evaluates the current literature comparing interlocking IMN to ORIF regarding radiographic and clinical outcomes.

## Materials and methods

### Study design and eligibility

This systematic review and meta-analysis adhered to the Preferred Reporting Items for Systematic Review and Meta-Analysis Protocols (PRISMA-P) Statement standards [16].

### Eligibility criteria

Studies that met the following criteria were considered eligible for inclusion: (1) study population age 18 and older, (2) patients with diaphyseal radius and/or ulna fractures, (3) studies comparing IMN versus ORIF, (4) patients were not concurrently treated with ORIF and IMN on the same fractured bone or ipsilateral forearm bones. Case series, reviews, letters, or commentaries were excluded. Only studies with an English manuscript were included.

### Search strategies

The MEDLINE and Embase databases were systematically searched for publications from January 1, 2000 to January 7, 2024. For MEDLINE, the following medical subject heading (Mesh) terms were used: “fracture fixation, intramedullary” OR “fracture fixation, internal” AND “radius fractures” OR “ulna fractures” OR “forearm injuries.” The Embase search included: (radius) OR (ulna) OR (both bone) AND (intramedullary) AND (internal fixation).

One author (M.W.B.) performed the search and excluded irrelevant articles and duplicates based on title and abstract. The remaining articles underwent an independent full-text review by two authors (M.W.B., M.E.W.) and were assessed for eligibility based on established criteria. Any conflicts were resolved by discussion.

### Data extraction

Baseline study information was collected, including the lead author, country of publication, and study design. Patient characteristics were also collected, including demographic information, fracture location and classification, operative times, and radiographic and clinical outcomes. The primary outcomes of interest were time-to-union, union rate, complication rate, patient-reported outcome scores (DASH and Grace–Eversmann scores), and supination and pronation range of motion (ROM). Secondary outcomes included operation time and radial bow. Outcomes were grouped based on treatment and then grouped into those reported for all forearm fractures, those reported for fractures involving both the radius and ulna simultaneously (i.e. both bone forearm fractures), and those reported fractures involving only the ulna. Meta-analysis for isolated radius fracture was unable to be performed due to only one study reporting these fractures.

### Risk of bias assessment and outcome quality appraisal

Non-randomized studies were evaluated using the Cochrane Risk Of Bias In Non-randomized Studies-of Interventions (ROBINS-I) tool [17]. Randomized controlled trials (RCTs) were evaluated using the Cochrane risk of bias (Rob) 2.0 tool for randomized controlled trials [18]. Two authors (M.W.B., M.E.W.) performed the bias assessment independently. Any disputes were settled through discussion.

The Grading of Recommendations, Assessment, Development, and Evaluations (GRADE) transparent framework was used to evaluate the certainty of evidence for each outcome. Outcomes were ranked as having high, moderate, low, or very low certainty [19].

### Sensitivity analysis

The leave-one-out method was used to assess the impact of individual studies. Resulting Baujat plots were used to identify studies effects on heterogeneity and effect size.

### Publication bias and heterogeneity between studies

Publication bias was assessed with funnel plots and the trim-and-fill method [20, 21]. Egger’s test was used to test the asymmetry of funnel plots.

### Statistical assessment

For cohort studies, an outcomes meta-analysis was performed comparing IMN and ORIF. The Mantel–Haenszel OR estimates were used for dichotomous variables. A standardized mean difference (SMD) was used to compare means with standard deviations. When the standard deviation was not reported but the sample range was, the standard deviation was estimated by dividing the range by four. Studies were included in forest plots if they reported zero total events to maintain analytic consistency. Heterogeneity was reported using the I2 statistic. A random effects model was used when the I2 statistic was over 50%, otherwise a fixed-effect model was used. The OR and SMD values were calculated with 95% confidence intervals (CI) and considered statistically significant if the 95% CI did not include 1 or 0, respectively. Meta-analysis was performed using Review Manager (RevMan, Version 5.4.1. Copenhagen: The Nordic Cochrane Centre, The Cochrane Collaboration, 2014).

## Results

### Study selection

**Fig. 1:**
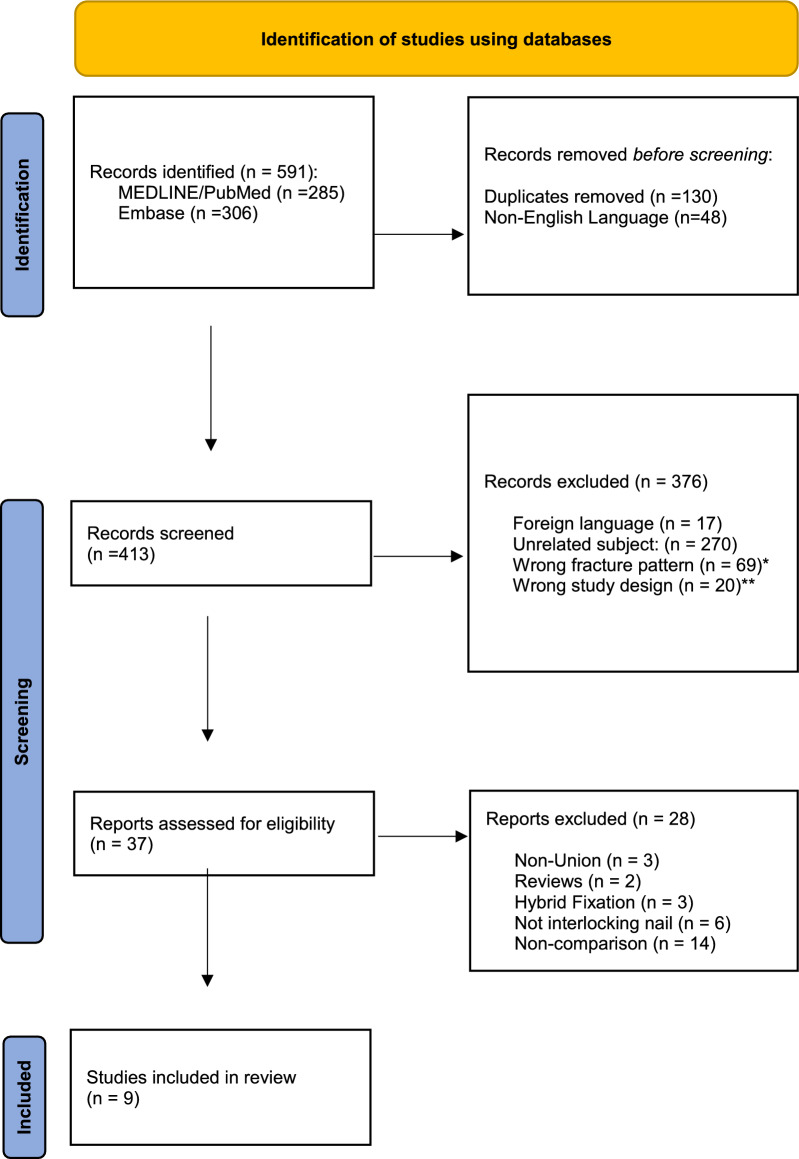
PRISMA flow diagram for the study search and selection (*Distal radius (n = 54), olecranon (n = 15). **Reviews/Case Reports/Technique Guide)

Database searches resulted in 591 records. After the removal of duplicate and non-English studies, 413 records remained. Abstract screening excluded 376 records, leaving 37 studies for full-text review. Twenty-eight further studies were excluded, leaving nine studies to be included in the meta-analysis (Fig. 1) [9, 22–29].

### Characteristics of included studies

Two randomized controlled trials (RCTs) and seven cohort studies met the inclusion criteria (Table 1) [9, 22–29]. There were 471 patients; 238 cases underwent IMN (51%), and 233 underwent ORIF (49%). Mean follow-up time ranged from 13 to 38 months. Two of the four groups in Zhang et al. [22] were excluded because they evaluated hybrid fixation (IMN of one bone and ORIF of the other).

**Table 1:** Characteristics of included studies

	Study design	Treatments	Treatment specifics	Patient number (n)	Mean Age ± SD (yrs)	Age range (yrs)	% Female	Mean follow-up ± SD (m)	Follow-up range (m)
Kibar and Kurtulmuş [28]	Retro. Cohort	IMN	TST	27	34.3	18–74	25.93	24	12–48
		ORIF	3.5 LC-DCP	22	36.8	17–68	13.64	29	
Kibar and Kurtulmuş [29]	Retro. Cohort	IMN	TST	27	31.3	18–67	25.93	21.6 ± 7.6	12–60
		ORIF	3.5 LC-DCP	30	46.2	23–78	40.00	29.8 ± 13.2	
Köse et al. [27]	Retro. Cohort	IMN	TST	48	36.6	18–63	22.92	14	13–42.5
		ORIF	3.5 LC-DCP	42	38.02	18–65	33.33	17.5	16–37.5
Lee et al. [9]	RCT	IMN	Acumed	35	43.1 ± 11	–	34.29	20	18–65
		ORIF	3.5 LCP	32	40.3 ± 10	–	31.25		
Ozkaya et al. [26]	Retro. Cohort	IMN	TST	20	33	18–70	30.00	23	12–34
		ORIF	3.5 LC-DCP	22	32	18–69	31.82	30	12–45
Pavone et al. [25]	Retro. Cohort	IMN	Acumed	9	47.2	22–83	33.33	12	–
		ORIF	LC-DCP	14	44.8	18–67	50.00		
Polat and Toy [24]	Retro. Cohort	IMN	TST	21	28.8	18–64	47.62	22.3	12–36
		ORIF	3.5 LC-DCP	25	32.4	19–97	36.00	24.8	12–48
Sisman and Polat [23]	Retro. Cohort	IMN	TST	29	39 ± 7.4	22–56	41.38	93**	84.5–99.5
		ORIF	3.5 LCP	25	36.3 ± 7.4		32.00	86**	80–97
Zhang et al. [22]*	RCT	IMN	Foresight®	22	37.8 ± 0.8	–	45.45	23.4	12–26
		ORIF	3.5 LCP	21	38.22 ± 1.15	–	42.86		

Retro, Retrospective; RCT, Randomized Controlled Trial; IMN, Intramedullary Nail; ORIF, Open Reduction and Internal Fixation; LCP, Locked Compression Plating; LC-DCP, Low Contact-Dynamic Compression Plating; wks, weeks; yrs, years; m, months

*2 groups excluded from analysis due to hybrid fixation (21 cases ulna ORIF and radius IMN, 23 cases, ulna IMN and radius ORIF) **Median

Overall, both-bone forearm fractures (BBFF) and AO/OTA type A fractures were the most commonly reported fracture types and classifications (Table 2) [9, 22–24, 27–29].

**Table 2:** Fracture characteristics of included studies

Study	Treatment	Fracture types			AO/OTA classification			Open fractures (%)
		Radius (%)	Ulna (%)	BBFF (%)	Type A (%)	Type B (%)	Type C (%)	
Both-bone forearm fractures								
Lee et al. [9]	IMN	0	0	100	46	54	0	26
	ORIF	0	0	100	47	53	0	31
Ozkaya et al. [26]	IMN	0	0	100	-*	-	-	5
	ORIF	0	0	100	-*	-	-	9
Polat and Toy [24]	IMN	0	0	100	43	43	14	38
	ORIF	0	0	100	44	36	20	36
Zhang et al. [22]	IMN	0	0	100	32	32	36	-**
	ORIF	0	0	100	38	24	38	-**
Isolated ulna fractures								
Kibar and Kurtulmuş [28]	IMN	0	100	0	52	41	7	7
	ORIF	0	100	0	73	20	7	7
Pavone et al. [25]	IMN	0	100	0	-	-	-	-**
	ORIF	0	100	0	-	-	-	-**
Sisman and Polat [23]	IMN	0	100	0	62	38	0	-**
	ORIF	0	100	0	76	24	0	-**
Isolated radius fractures								
Kibar and Kurtulmuş [29]	IMN	100	0	0	78	22	0	15
	ORIF	100	0	0	68	27	5	5
Mixed fractures								
Köse et al. [27]	IMN	31	31	38	38	31	31	25
	ORIF	33	29	38	36	36	29	19
Overall [n(%)]^†^								
	IMN	42 (17%)	80 (34%)	116 (49%)	102 (43%)	77 (32%)	30 (12%)	
	ORIF	36 (15%)	81 (35%)	116 (50%)	104 (45%)	64 (27%)	29 (12%)	

*A3 was most common, breakdown not reported; **Excluded open fractures; ^†^29 fractures not classified by the AO classification with IMN; 36 not classified with ORIF (BBFF, Both-bone Forearm Fractures; IMN, Intramedullary Nail; ORIF, Open Reduction and Internal Fixation)

### Risk of bias assessment

Four of seven non-randomized studies had a moderate risk of bias with the ROBINS-I tool due to surgeons choosing treatment based on preference (Supplementary File 1, Appendix A: Table 1) [24, 25, 28, 29]. Ozkaya et al. [26], Köse et al. [27], and Sisman and Polat [23] were at serious risk of bias due to bias in selection, bias in selection and intervention deviation, and bias due to missing data, respectively. Two RCTs were evaluated as having a low risk of bias using Rob 2.0 analysis with some concern due to the inability to blind participants and providers to the treatment (Supplementary File 1, Appendix A: Fig. 1) [9, 22].

## Evaluation of outcomes

### All fracture types

Appendix B (Supplementary File 2) summarizes each study that evaluated each outcome for all fractures, BBFF, and isolated ulna fractures, whether included in the meta-analysis or not.

### Operative time, complications, and implant removal

**Fig. 2:**
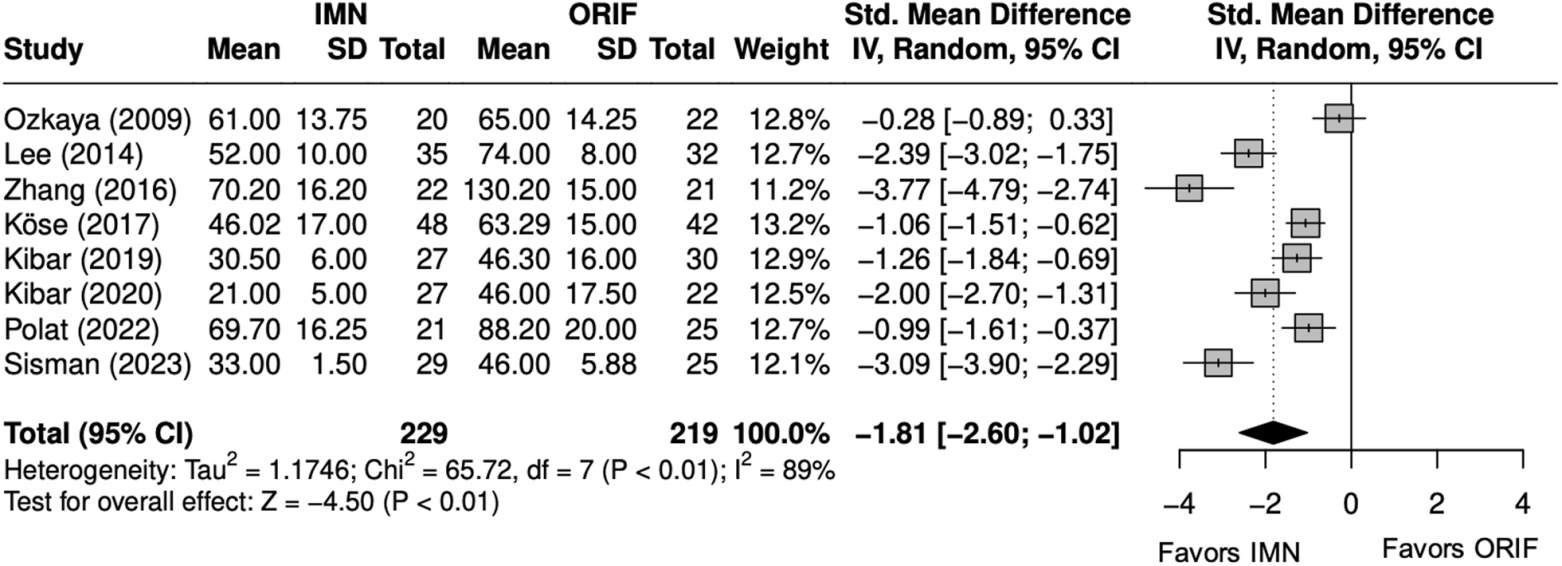
Forest plot of operative time meta-analysis for all fracture types

**Fig. 3:**
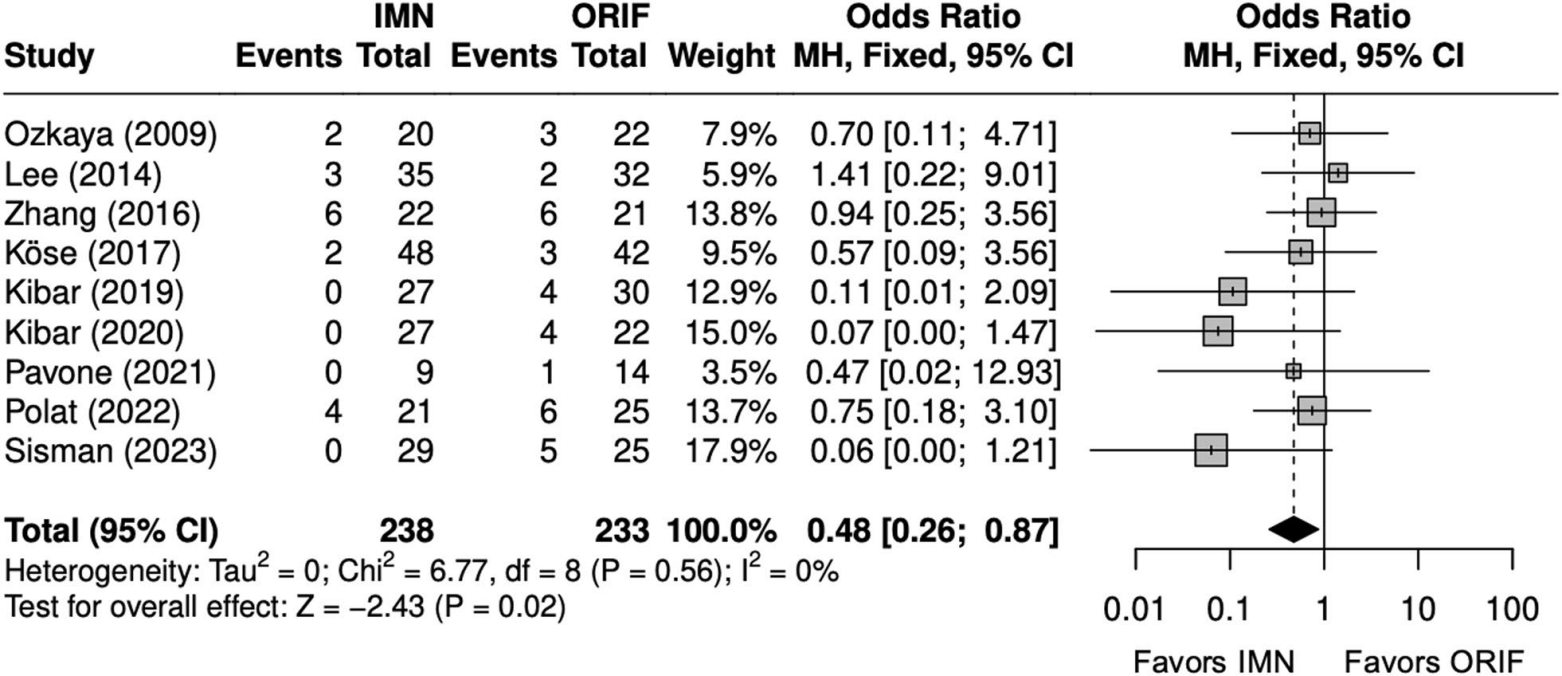
Forest plot of complications meta-analysis for all fracture types

**Fig. 4:**
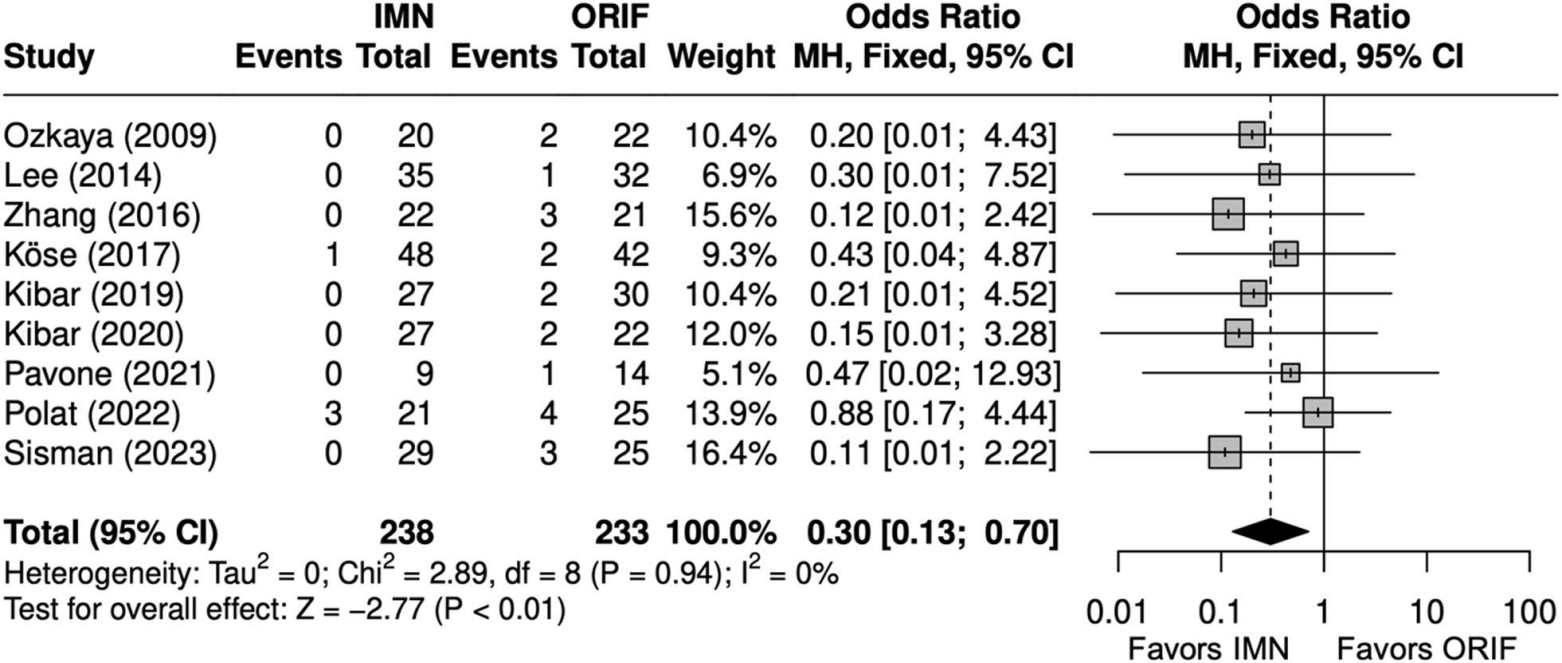
Forest plot of surgical site infection meta-analysis for all fracture types

**Fig. 5:**
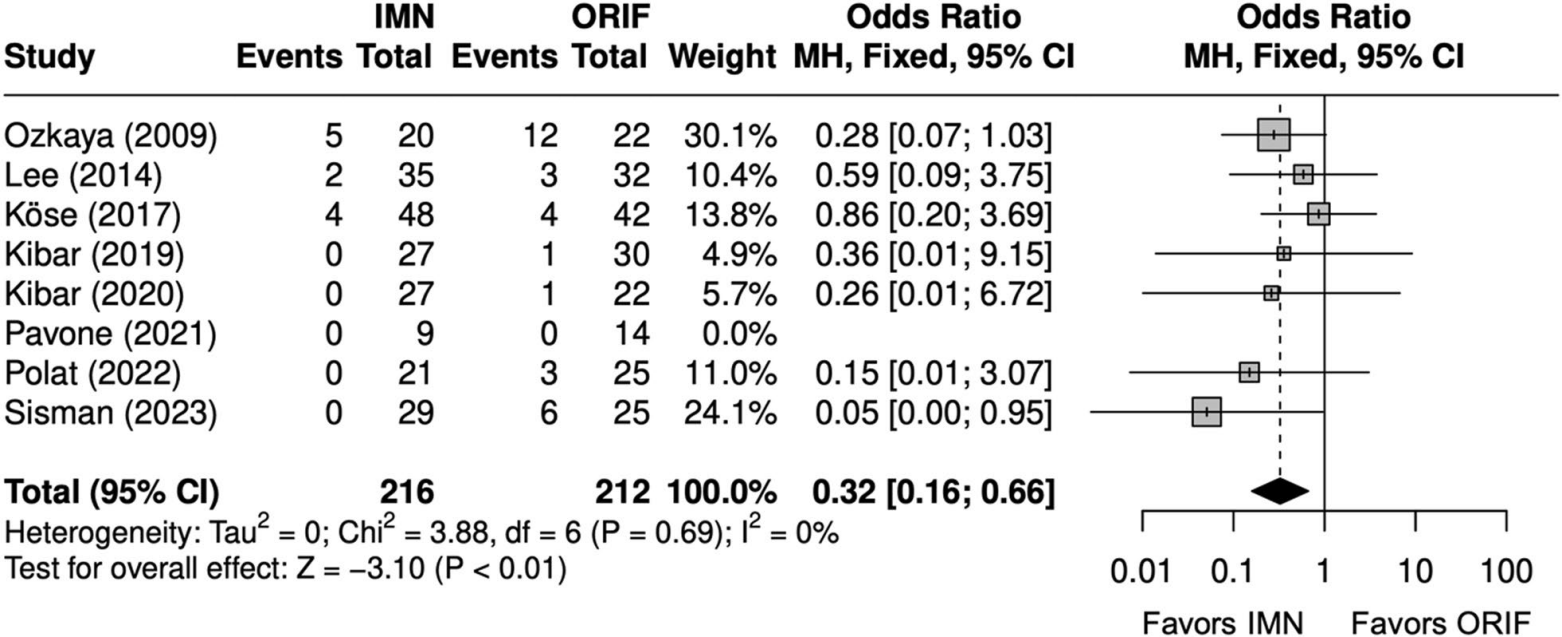
Forest plot of implant removal meta-analysis for all fracture types

Operative time (minutes) was significantly shorter with IMN (SMD = − 2 [− 3, − 1]) (Fig. 2) [9, 22, 24–29]. All studies reported complication rates, which included nonunion, delayed union, malunion, nerve injury, surgical site infection (SSI), and extensor pollicis longus (EPL) tendon rupture. The complication rate and SSI rate were significantly lower with IMN (OR = 0.48 [0.26, 0.87]) (Fig. 3), (OR = 0.30 [0.13, 0.71]) (Fig. 4). [9, 22–29] The implant removal rate was significantly lower with IMN in all forearm fractures (OR = 0.33 [0. 16, 0.66]) (Fig. 5). [9, 23–29]

### Radiographic outcomes

Postoperative immobilization protocols varied across studies and are described in Table 3 [9, 22–29].

**Table 3:** Post-operative immobilization protocols

	Immobilization IMN	Immobilization ORIF
Both-bone forearm fractures		
Lee et al. [9]	STS: 2 weeks + 4 weeks elbow brace w/neutral wrist	None
Ozkaya et al. [26]	Secure Distal 2/3: None	None
	Secure Prox. 1/3: Cast/orthosis: 2–3 weeks	
	Not secure: LAC: Until callus formation	
Polat and Toy [24]	None	LAC: 2 weeks
Zhang et al. [22]	LAS- 2 weeks	None
Isolated ulna fractures		
Kibar and Kurtulmuş [28]	None	LAC: 2–3 weeks
Pavone et al. [25]	None	STS: 2 weeks
Sisman and Polat [23]	None	None
Isolated radius fractures		
Kibar and Kurtulmuş [29]	None	LAC: 2–3 weeks
Mixed Fractures		
Köse et al. [27]	None	STS: 2 weeks

IMN, Intramedullary Nail; ORIF, Open Reduction and Internal Fixation LAC, Long-arm cast; STS, Sugar Tong Splint; Prox., Proximal; LAS, Long-arm Splint

**Fig. 6:**
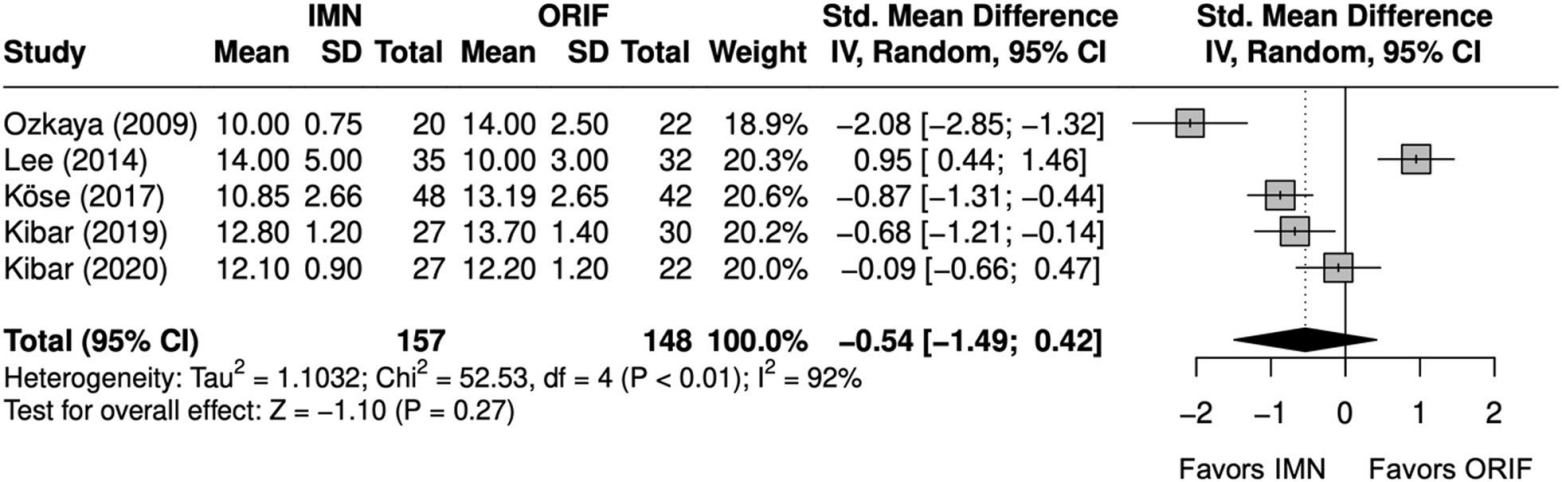
Forest plot of time-to-union meta-analysis for all fracture types

**Fig. 7:**
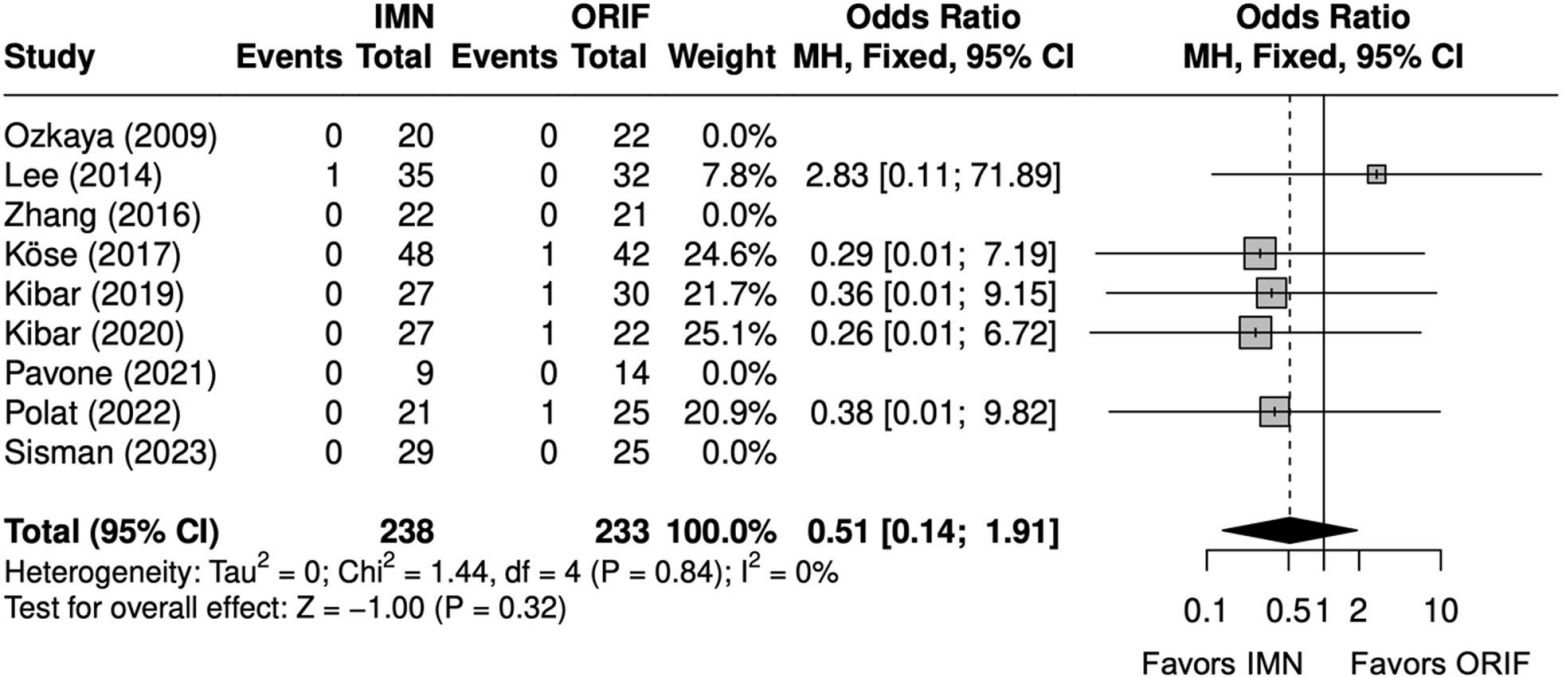
Forest plot of nonunion meta-analysis for all fracture types

Time-to-union (weeks) was compared in five studies [9, 26–29]. Three studies were excluded from the meta-analysis; all reporting significantly shorter time-to-union with IMN. Zhang et al. [22] and Polat et al. [24] reported mean time-to-union without an associated SD or range, and Pavone et al. [25] reported time-to-union by time interval. Time-to-union in the meta-analysis was similar between IMN and ORIF (SMD = − 0.5 [− 1.5, 0.5]), with significant heterogeneity (I^2^ = 93%) (Fig. 6). The nonunion rate was similar between IMN and ORIF (OR = 0.51 [0.14, 1.92]) (Fig. 7) [9, 22–29].

### Functional outcomes

#### DASH

**Fig. 8:**
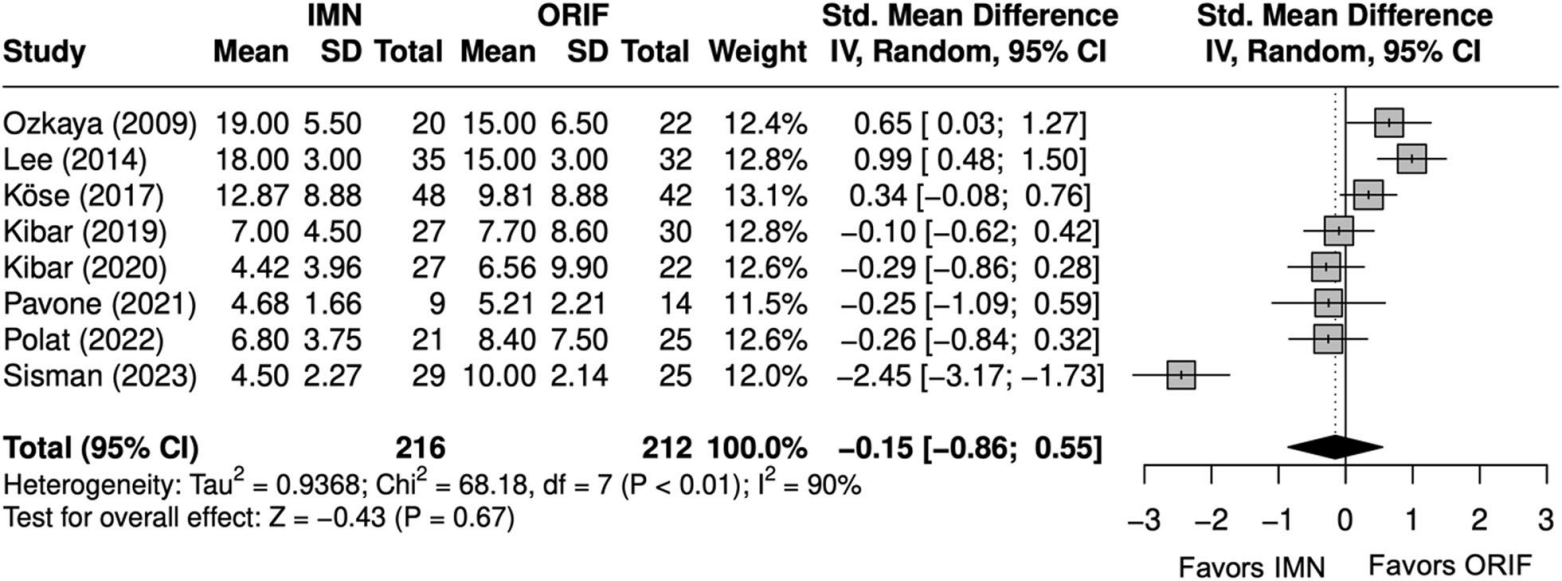
Forest plot of DASH meta-analysis for all fracture types

**Fig. 9:**
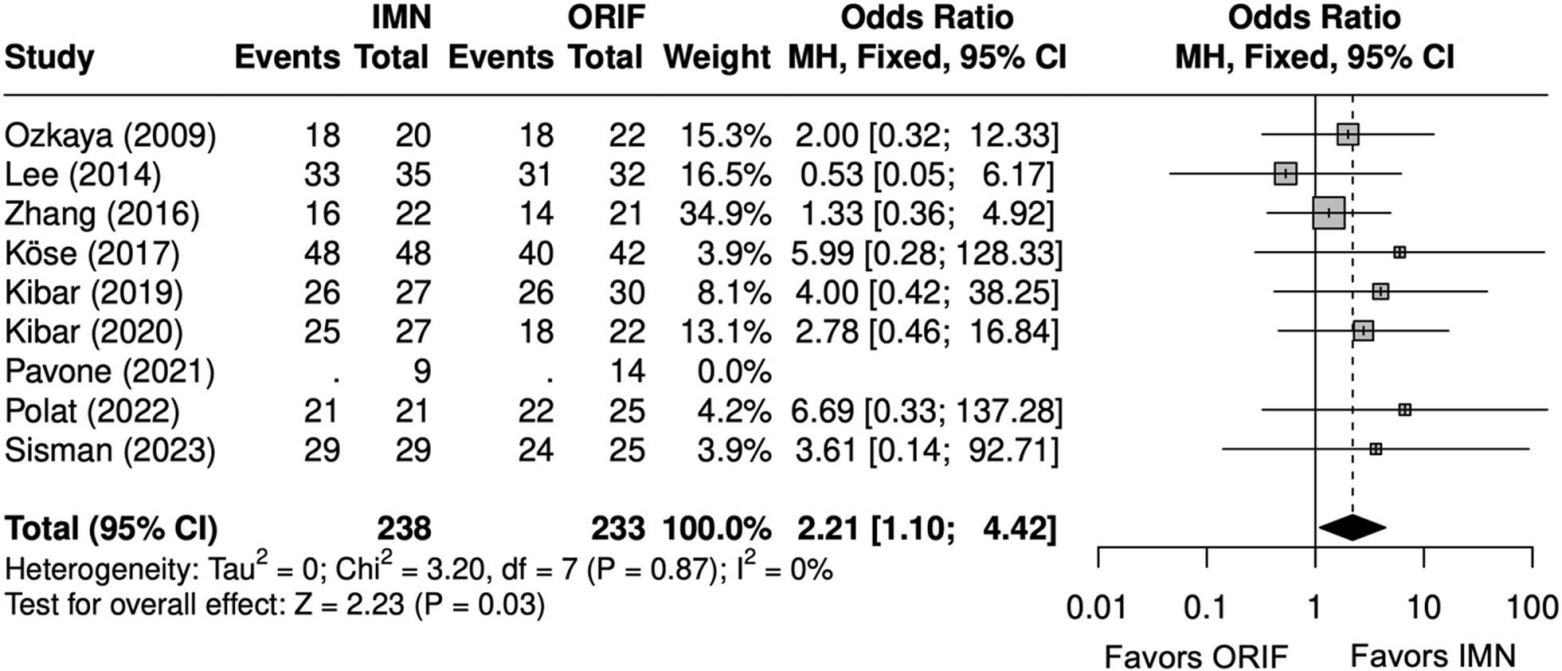
Forest plot of Grace–Eversmann score meta-analysis for all fracture types

**Fig. 10:**
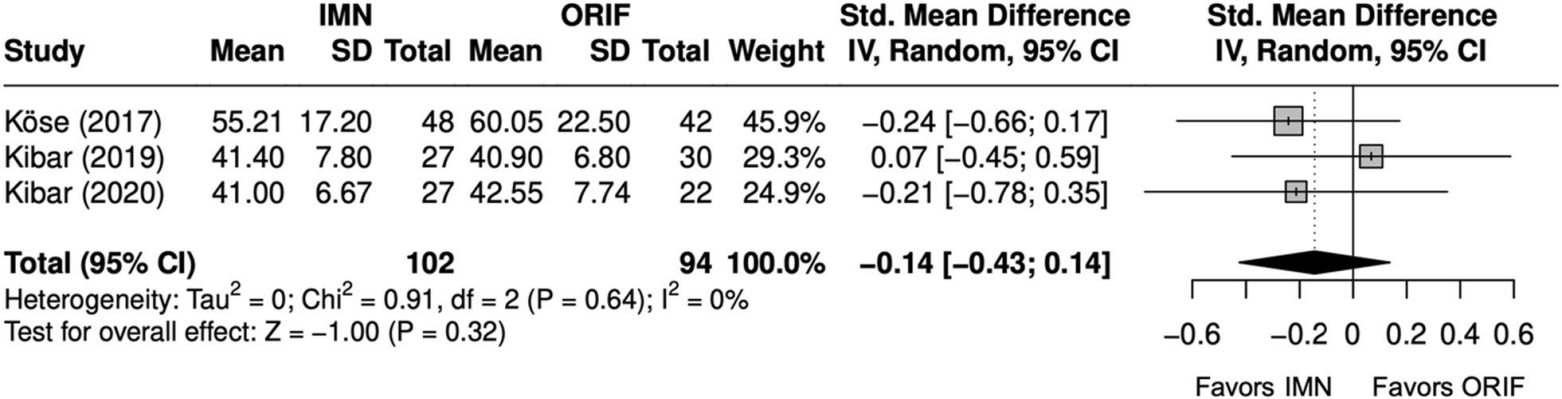
Forest plot of grip strength score meta-analysis for all fracture types

Scores were similar between IMN and ORIF overall (SMD = − 0.2 [− 1, 1]) (Fig. 8) [9, 23–29]. Excellent and good Grace–Eversmann scores were more likely to occur with IMN overall (OR = 2.2 [1.1, 4.4]) (Fig. 9) [9, 22–24, 26–29]. Pronosupination ROM (degrees) was similar between IMN and ORIF (SMD = 0.4 [− 2.4, 3.1]) [9, 24, 27–29]. Grip strength (kg) was similar between IMN and ORIF (SMD = − 0.1 [− 0.4, 0.1]) (Fig. 10) [27–29].

### Both-bone forearm fractures

#### Operative time, complications, and implant removal

**Fig. 11:**
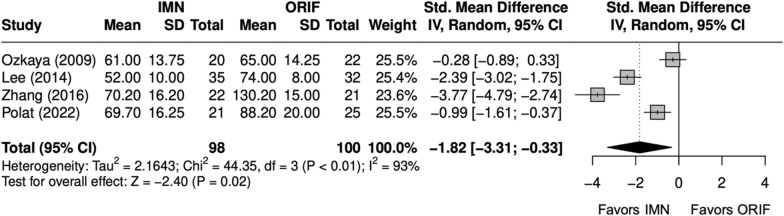
Forest plot of operative time meta-analysis for both bone forearm fracture studies

**Fig. 12:**
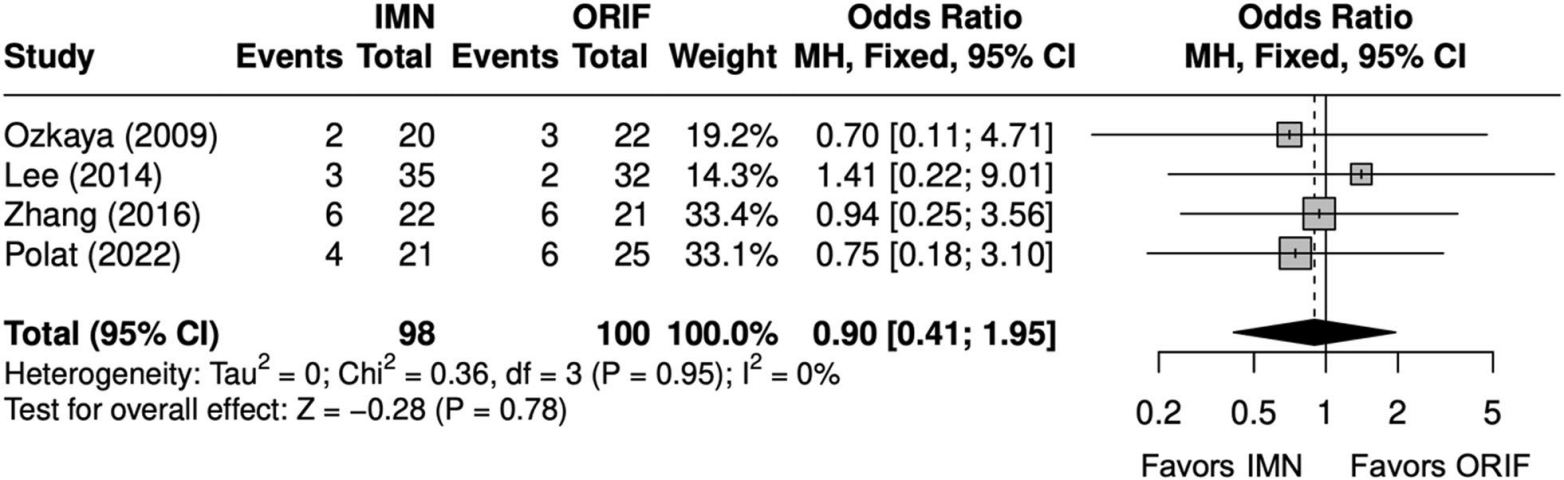
Forest plot of complications meta-analysis for both bone forearm fracture studies

**Fig. 13:**
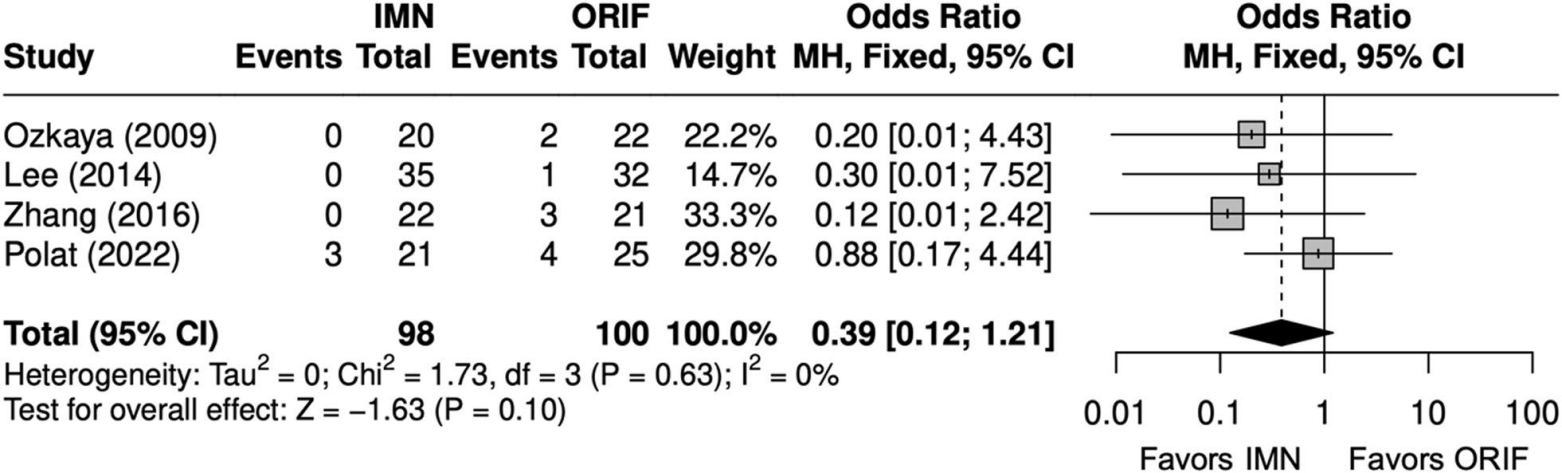
Forest plot of surgical site infection meta-analysis for both bone forearm fracture studies

**Fig. 14:**
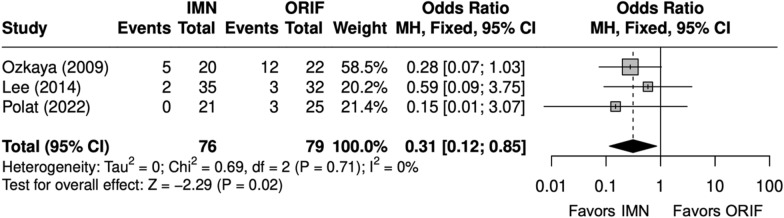
Forest plot of implant removal meta-analysis for both bone forearm fracture studies

Overall, four studies evaluated BBFF [9, 22, 24, 26]. Operative time (minutes) was shorter with an IMN (SMD = 2 [− 3, − 0.3]) (Fig. 11) [9, 22, 24, 26]. The complication rate and SSI rate were similar between IMN and ORIF (OR = 0. 90 [0.41, 1.95]) (Fig. 12), (OR = 0. 39 [0. 12, 1. 21]) (Fig. 13) [9, 22, 24, 26]. The implant removal rate was compared in three BBFF studies [9, 24, 26]. The implant removal rate was significantly lower with IMN (OR = 0.31 [0.12, 0.85]) (Fig. 14) [9, 24, 26].

### Radiographic outcomes

**Fig. 15:**
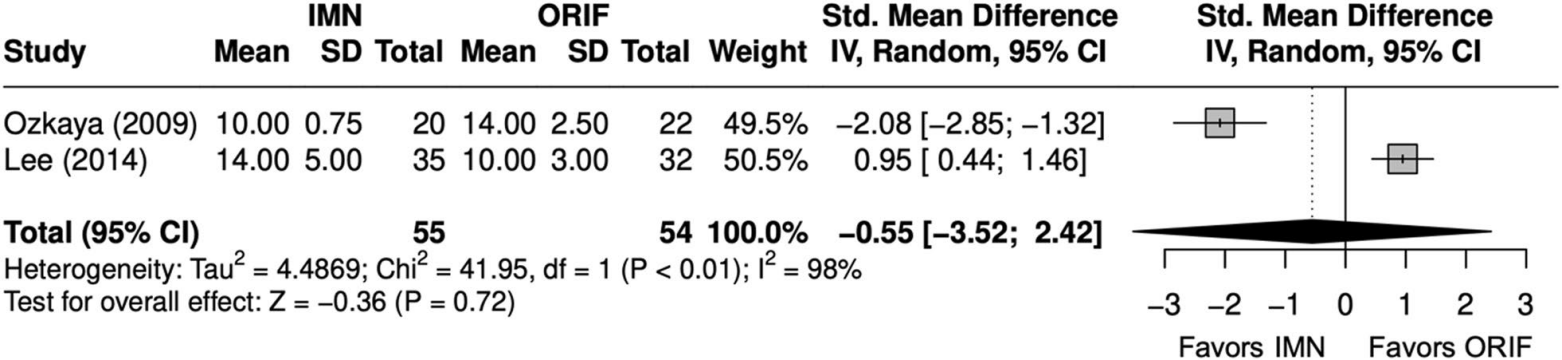
Forest plot of time-to-union meta-analysis for both bone forearm fracture studies

**Fig. 16:**
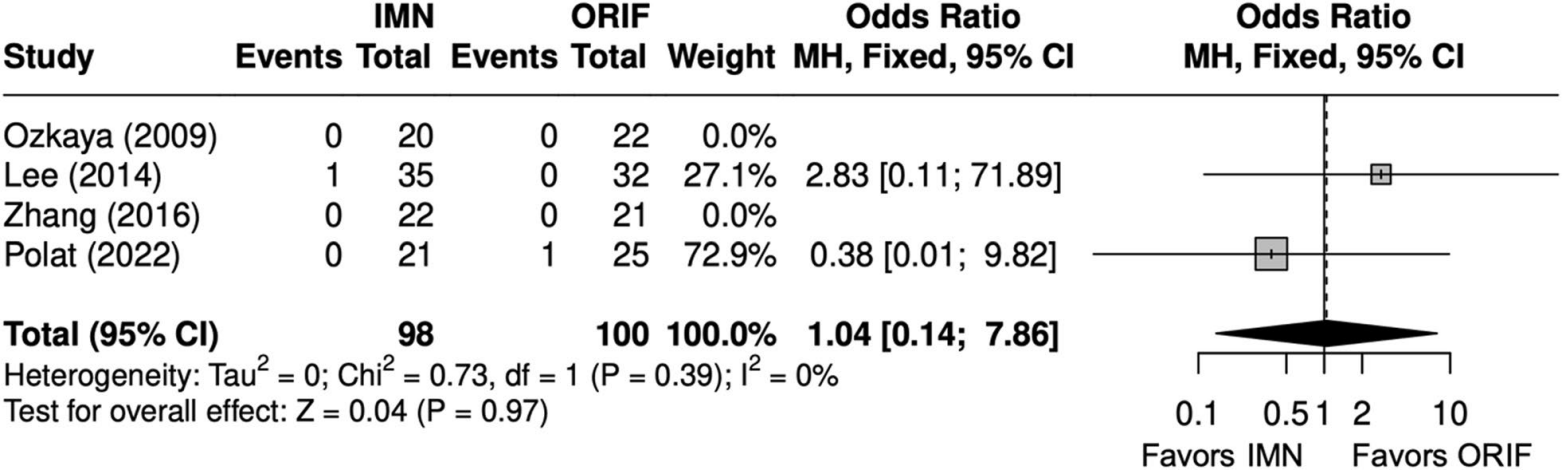
Forest plot of nonunion meta-analysis for both bone forearm fracture studies

Time-to-union (weeks) was similar between IMN and ORIF in BBFF (SMD = − 0.6 [− 3.5, 2.4]), with significant heterogeneity (I^2^ = 98%) (Fig. 15) [9, 26]. This was the only outcome with a very low certainty GRADE primarily due to imprecision and inconsistency of results. The nonunion rate was similar between IMN and ORIF in BBFF (OR = 1.04 [0.14, 7.86]) (Fig. 16) [9, 22, 24, 26].

### Functional outcomes

**Fig. 17:**
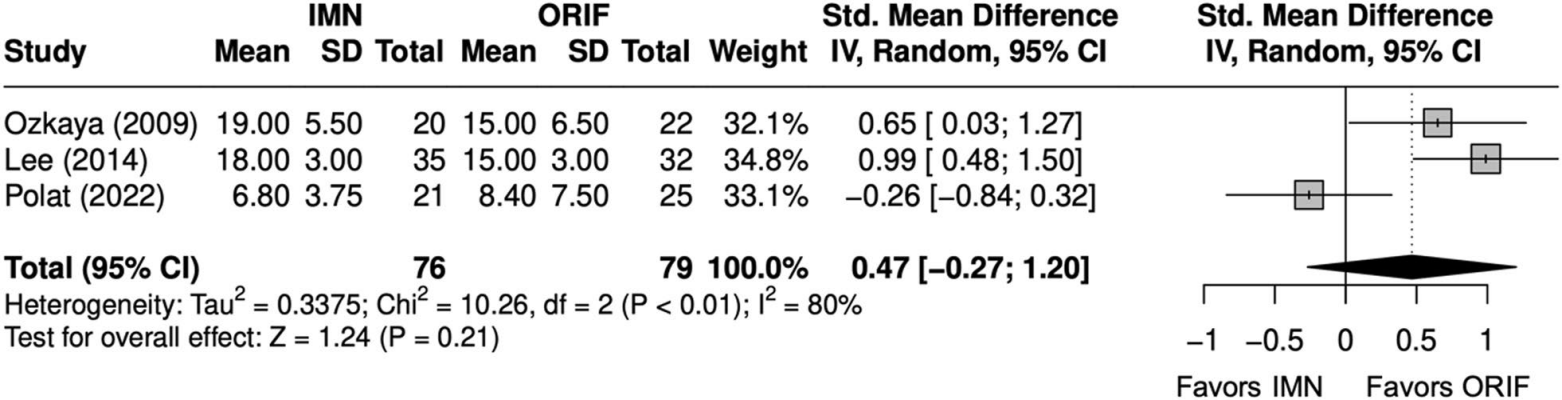
Forest Plot of DASH meta-analysis for both bone forearm fracture studies

**Fig. 18:**
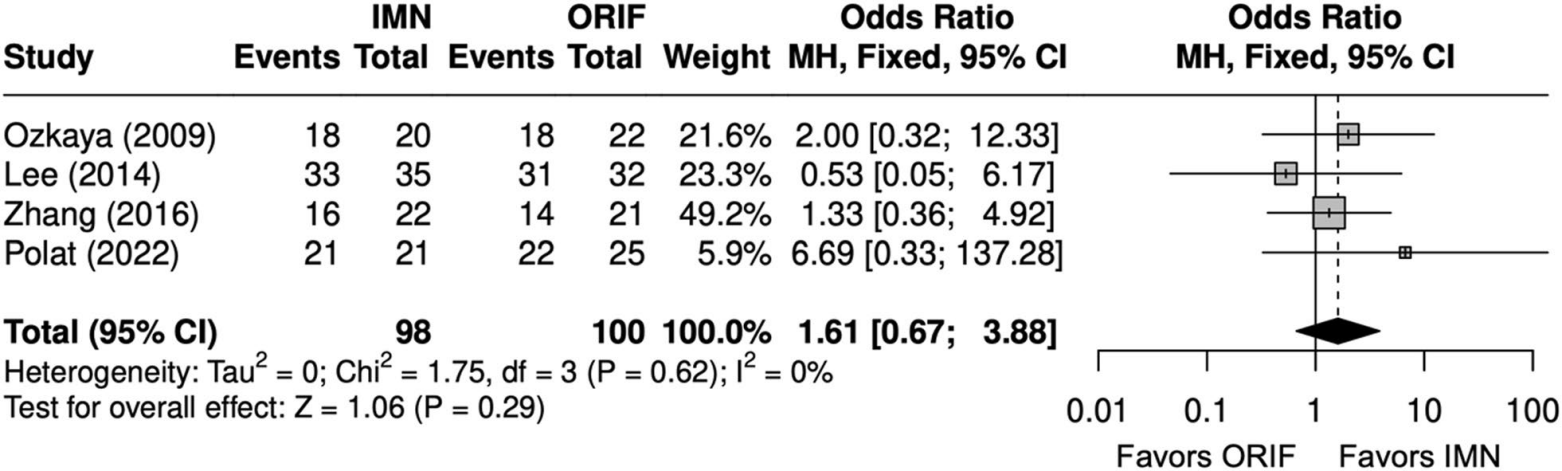
Forest plot of Grace–Eversmann scores meta-analysis for both bone forearm fracture studies

DASH scores were similar between IMN and ORIF (SMD = 0.5 [− 0.3, 1]) (Fig. 17) [9, 23–29]. Excellent and good Grace–Eversmann scores occurred at a similar rate, with IMN in BBFF (OR = 1.61 [0.67, 3.88]) (Fig. 18) [9, 22, 24, 26].

### Isolated ulna fractures

#### Operative time, complications, and implant removal

**Fig. 19:**
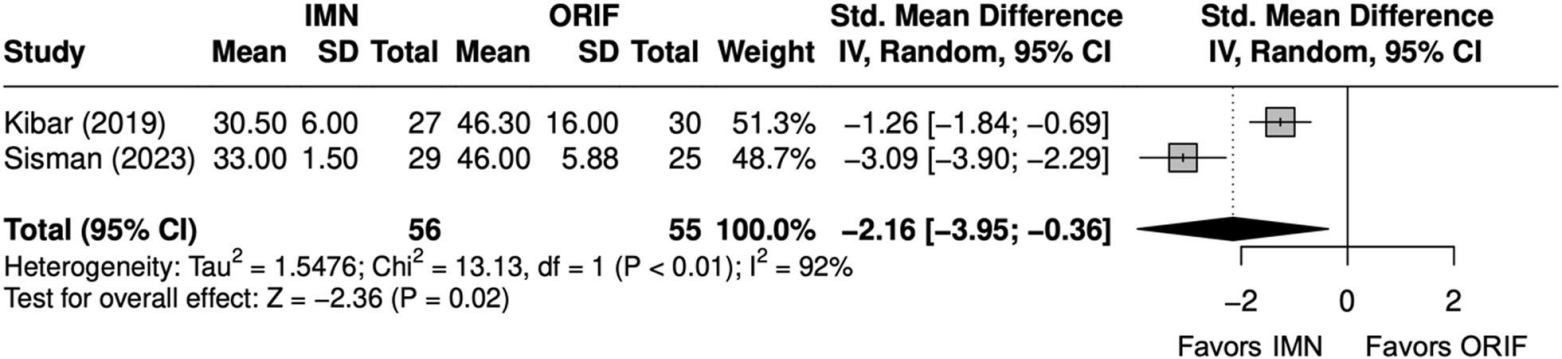
Forest plot of operative time meta-analysis for ulna fracture studies

**Fig. 20:**
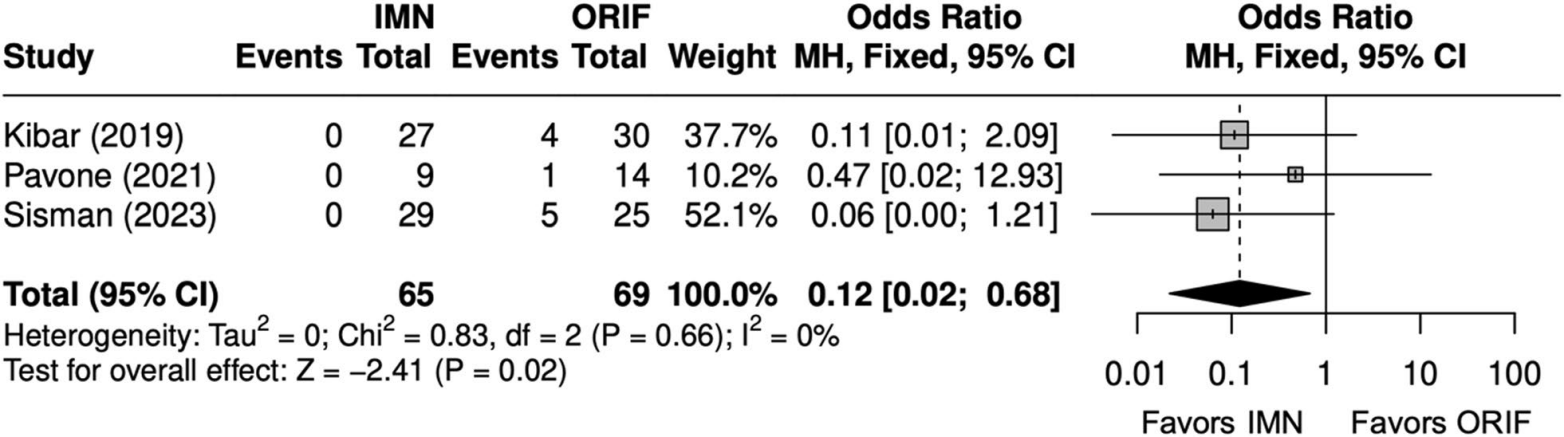
Forest plot of complication meta-analysis for ulna fracture studies

**Fig. 21:**
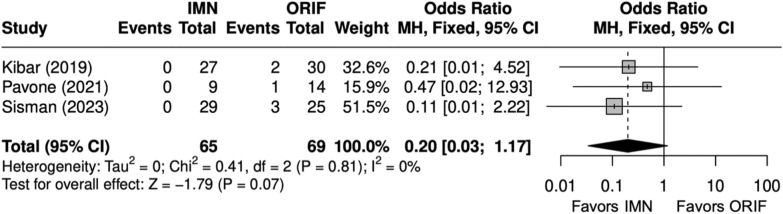
Forest plot of surgical site infection meta-analysis for ulna fracture studies

**Fig. 22:**
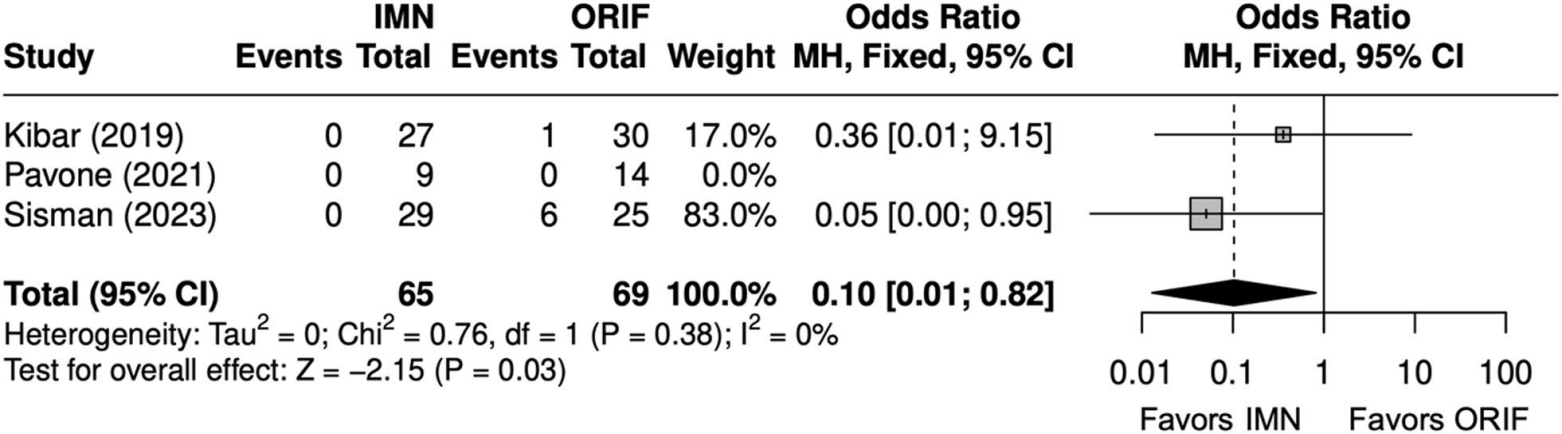
Forest plot of implant removal meta-analysis for ulna fracture studies

Overall, three studies evaluated isolated ulna fractures [23, 25, 29]. Operative time (minutes) was significantly shorter for IMN (SMD = − 2 [− 4, − 0.4]) (Fig. 19). Complication rate was significantly shorter for IMN (OR = 0.12 [0.02, 0.68]) (Fig. 20). SSI was not significantly different between IMN and ORIF (OR = 0.20 [0.03, 1.17]) (Fig. 21). IMN had a significantly decreased risk of implant removal (OR = 0.10 [0.01, 0.82]) (Fig. 22).

### Radiographic outcomes

No meta-analysis was possible for radiographic outcomes in isolated ulna fractures as only one study [29] was included in evaluating time to union and had zero nonunions.

#### Functional outcomes

**Fig. 23:**
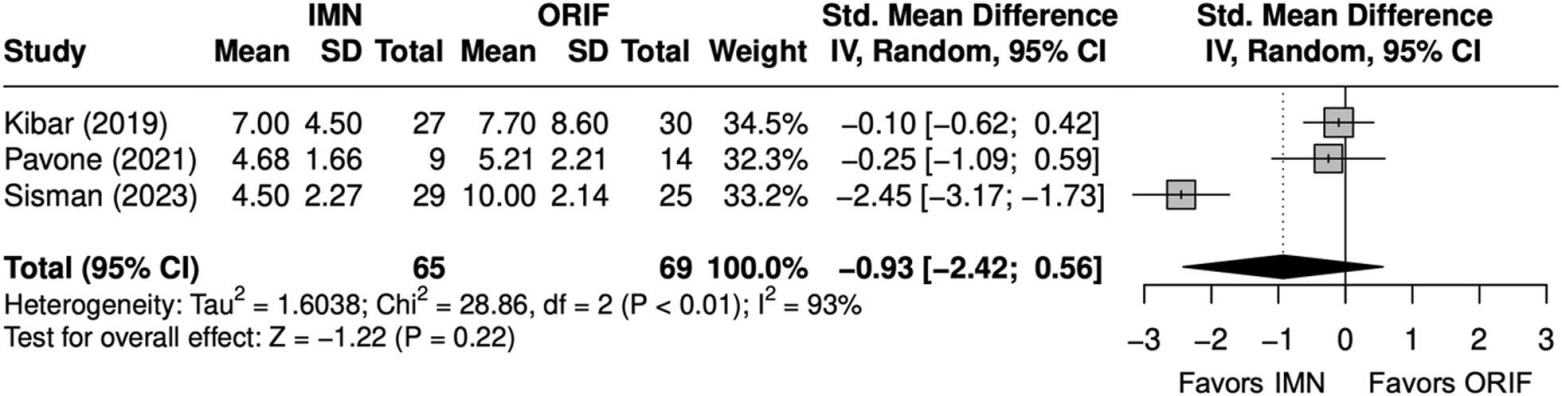
Forest plot of DASH meta-analysis for ulna fracture studies

**Fig. 24:**
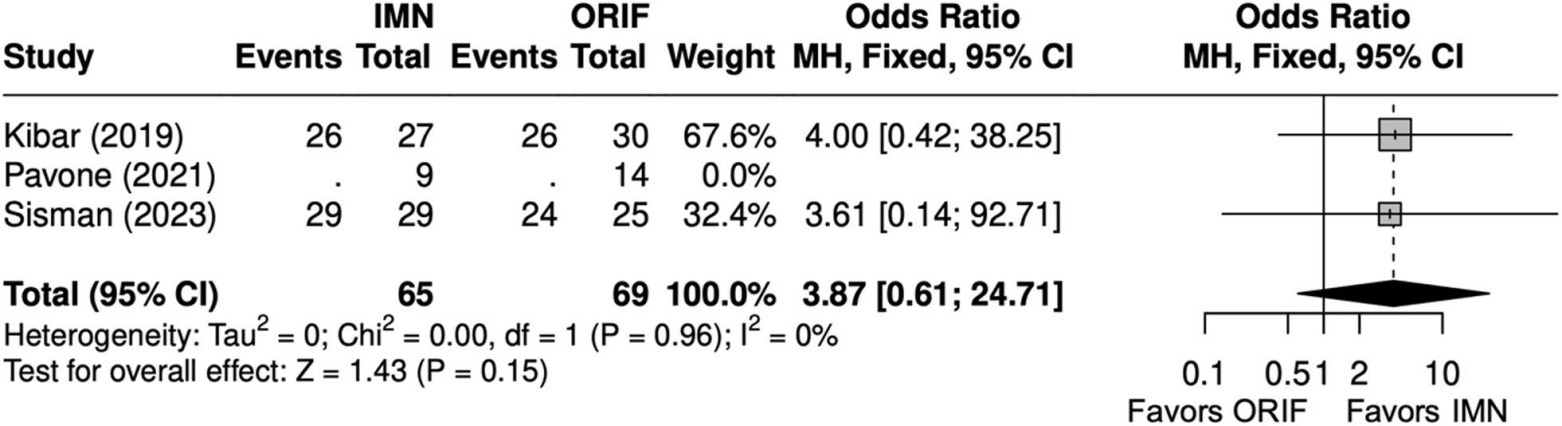
Forest plot of Grace–Eversmann scores meta-analysis for ulna fracture studies

DASH scores were similar between IMN and ORIF (SMD = − 1 [− 2, 1] (Fig. 23). Excellent or good Grace–Eversmann scores were more likely to occur with IMN than ORIF but were not significantly different (OR = 3.9 [0.6, 24.7]) (Fig. 24).

#### Sensitivity analysis and publication bias

Sensitivity analysis using the leave-one-out method and resulting Baujat plots are detailed in Appendix C (Supplementary File 3). Complication rates were the only outcomes that became not significant when excluding Şişman et al. [23], however excluding Zhang et al. [22] made the effect more in favor of IMN. The leave-one-out method influenced Grace–Eversmann score outcomes the least.

Publication bias using funnel plots, the trim-and-fill method, and Egger's test are detailed in Appendix D (Supplementary File 4). Moderate impact of potential publication bias was found with complications, surgical site infections, and Grace–Eversmann scores outcomes (Table 4).

**Table 4:** Potential impact of publication bias for all outcomes

Outcome	Potential Publication Bias Impact	Comment
Operative time	Minimal	The effect size remains significant after adjustment, suggesting limited influence of bias on observed heterogeneity
Complications	Moderate	Initial findings may have been overestimated due to bias, as indicated by the non-significant effect after adjustment
Surgical site infection	Moderate	Initial effect size was influenced by bias, but the result remained non-significant after adjustment
Implant removal	Minimal	The significant effect persists even after adjusting for bias, indicating limited effect of bias on results
Time-To-Union	Minimal	Bias had little impact on the non-significant result
Nonunion Rates	Minimal	Publication bias does not substantially affect the non-significant result
DASH scores	Minimal	Publication bias does not substantially affect the non-significant result
Grace–Eversmann scores	Moderate	Initial significant effect was influenced by bias, with adjustment showing a non-significant effect

#### GRADE criteria and result summary

Table 5 summarizes the meta-analysis results and GRADE criteria for all fractures, BBFF, and isolated ulna fractures.

**Table 5:** Summary of outcomes and GRADE criteria for all outcomes measured by meta-analysis

Interlocking intramedullary nail compared to open reduction and internal fixation for forearm diaphyseal fractures									
Patient or population: Adults with diaphyseal forearm fractures									
Settings: Acute injury									
Intervention: Intramedullary nail									
Comparison: Open reduction and internal fixation									
Outcomes (studies)	Total IMN (events)	Total ORIF (events)	Standardized Mean Difference//Odds ratio (95% CI)	Chi^2^ (*P*-value)	I^2^	Z (*P*-value)	Result	Certainty of the evidence (GRADE)	Comment
Operative time (minutes)									
All Fractures (8)	200	194	SMD − 2 [− 3.6, − 1.9]	65.72 (*P* < 0.01)	89%	− 4.50 (*P* < 0.01)	IMN has shorter operation times than ORIF	Low	Imprecision; Observational Data; Statistically, but not clinically shorter
BBFF (4)	98	100	SMD − 2 [− 3, − 0. 3]	44.35 (*P* < 0.01)	93%	− 2.40 (*P* = 0.02)	IMN has shorter operation times than ORIF	Low	Imprecision; Observational Data; Statistically, but not clinically shorter
Ulna (2)	56	55	SMD = − 2 [− 4, − 0.4]	13.13 (*P* < 0.01;	92%	− 2.36 (*P* = 0.02)	IMN has shorter operation times than ORIF	Low	Imprecision; Observational Data; Statistically, but not clinically shorter
Complications									
Complications									
All Fractures (9)	238 (17)	233 (34)	OR = 0.5 [0.26, 0.87]	6.77 (*P* = 0.56)	0%	− 2.43 (*P* = 0.02)	IMN has lower complication rates than ORIF	Low	Observational Data
BBFF (4)	98 (15)	100 (17)	OR = 0.9 [0.41, 1.95]	− 0.28 (*P* = 0.78)	0%	− 0.28 (*P* = 0.78)	IMN has similar complication rates to ORIF	Low	Observational Data
Ulna (3)	65 (0)	69 (10)	OR = 0.12 [0.02, 0.68]	0.83 (*P* = 0.66)	0%	− 2.41 (*P* = 0.02)	IMN has lower complication rates than ORIF	Low	Observational Data
Surgical site infection									
All Fractures (9)	238 (4)	233 (20)	OR = 0.3 [0.13, 0.71]	2.89 (*P* = 0.94)	0%	− 1.63 (*P* = 0.10)	IMN has lower SSI rates than ORIF	Low	Observational Data
BBFF (4)	98 (3)	100 (10)	OR = 0. 39 [0. 12, 1. 21]	1.73 (*P* = 0.63)	0%	1.63 (*P* = 0.10)	IMN has similar SSI rates to ORIF	Low	Observational Data
Ulna (3)	65 (0)	69 (6)	OR = 0.1 [0.01, 0.82]	0.41 (*P* = 0.811	0%	− 1.79 (*P* = 0.07)	IMN has similar SSI rates to ORIF	Low	Observational Data
Implant removal									
All Fractures (8)	216 (8)	212 (27)	OR = 0.33 [0. 16, 0.66]	3.88 (*P* = 0.69)	0%	− 3.10 (*P* < 0.01)	IMN has lower implant removal rates than ORIF	Low	Observational Data
BBFF (3)	76 (7)	79 (18)	OR = 0.31 [0.12, 0.85]	0.69 (*P* = 0.71)	0%	− 2.29 (*P* = 0.02)	IMN has lower implant removal rates than ORIF	Low	Observational Data
Ulna (3)	65 (0)	69 (7)	OR = 0.1 [0.01, 0.82]	0.76 (*P* = 0.38)	0%	− 2.15 (*P* = 0.03)	IMN has lower implant removal rates than ORIF	Low	Observational Data
Radiographic outcomes									
Time-to-Union (weeks)									
All Fractures (5)	157	148	SMD = − 1 [− 1.6, − 0.3]	52.53 (*P* < 0.01)	92%	− 1.10 (*P* = 0.27)	IMN has similar time-to-union as ORIF	Low	Inconsistency; Imprecision; Observational Data
BBFF (2)	55	54	SMD = − 1 [− 3.5, 2.4]	41.95 (*P* < 0.01)	98%	− 0.36 (*P* = 0.72)	IMN has similar time-to-union as ORIF	Very Low	Inconsistency; Imprecision; Observational Data
Nonunion rate									
All Fractures (9)	238 (4)	233 (9)	OR = 0.51 [0.14, 1.92]	1.44 (*P* = 0.84)	0%	− 1.00 (*P* = 0.32)	IMN has similar nonunion rate as ORIF	Low	Imprecision; Observational Data
BBFF (4)	98 (4)	100 (4)	OR = 1.04 [0.14, 7.86]	0.73 (*P* = 0.39)	0%	0.04 (*P* = 0.97)	IMN has similar nonunion rate as ORIF	Low	Imprecision; Observational Data
Functional outcomes									
DASH score									
All Fractures (8)	216	212	SMD = − 0.2 [− 1, 1]	68.18 (*P* < 0.01)	90%	− 0.43 (*P* = 0.67)	IMN has similar DASH scores as ORIF	Low	Inconsistency; Imprecision; Observational Data
BBFF (3)	76	79	SMD = 0.5 [− 0.3; 1]	10.26 (*P* < 0.01)	80%	1.24 (*P* = 0.21)	IMN has similar DASH scores as ORIF	Low	Observational Data
Ulna (3)	65	69	SMD = − 1 [− 2, 1]	28.86 (*P* < 0.01)	93%	− 1.22 (*P* = 0.22)	IMN has similar DASH scores as ORIF	Low	Observational Data
Excellent or good Grace–Eversmann score									
All Fractures (8)	219 (207)	209 (183)	OR = 2.2 [1.1, 4.4]	3.20 (*P* = 0.87)	0%	2.23 (*P* = 0.03)	IMN has improved GE scores to ORIF	Low	Observational Data
BBFF (4)	88 (79)	90 (75)	OR = 1.6 [0.7, 3.9]	1.75 (*P* = 0.62)	0%	1.06 (*P* = 0.29)	IMN has similar GE scores as ORIF	Low	Observational Data
Ulna (2)	65 (55)	69 (50)	OR = 3.9 [0.6, 24.7]	0.00 (*P* = 0.96)	0%	1.43 (*P* = 0.15)	IMN has similar GE scores as ORIF	Low	Observational Data
Pronosupination ROM (^o^)									
All Fractures (5)	158	151	SMD = 0.4 [− 2.4, 3.]	1.49 (*P* = 0.83)	0%	0.28 (*P* = 0.78)	IMN has similar ROM as ORIF	Low	Observational Data
BBFF (2)	56	57	SMD = − 0.3 [− 4.5, 4.0]	1.19 (*P* = 0.28)	0%	0.13 (*P* = 0.90)	IMN has similar ROM as ORIF	Low	Imprecision; Observational Data
Grip Strength (kg)									
All Fractures (3)	102	94	SMD − 0.1 [− 0.4, 0.1]	0.91 (*P* = 0.63)	0%	− 1.00 (*P* = 0.32)	IMN has similar grip strength as ORIF	Low	Imprecision; Observational Data

BBFF, Both-bone forearm fracture; IMN, Intramedullary Nail; ORIF, Open Reduction and Internal Fixation; SMD, Standardized Mean Difference; OR, Odds Ratio, ROM, Range of motion; SSI, Surgical Site Infection; GE, Grace–Eversmann

## Discussion

This systematic review and meta-analysis aimed to compare radiographic and functional outcomes of interlocked IMN fixation to ORIF for forearm diaphyseal fractures in adults. For all studies, the operative time, overall complication rate, SSI rate, and implant removal rates were lower with IMN than with ORIF. Union rates were similar between IMN and ORIF, but the time-to-union trended towards shorter with IMN. “Excellent” and “good” Grace–Eversmann scores were higher with IMN than ORIF. DASH scores, range of pronosupination, and grip strength were similar between IMN and ORIF. Subgroup analysis of isolated ulna fractures and BBFF showed that operative time and implant removal rate remained significantly lower with IMN than with ORIF. Overall complication rates remained significantly lower with IMN in isolated ulna fractures. Otherwise, all outcomes were similar between IMN and ORIF.

Operation time and blood loss were found to be significantly lower with IMN. ORIF operative times were consistent with previous literature [30]. Although IMN had a statistically significant lower operative time, a difference of 2 min is not clinically significant. IMN requires increased reliance on fluoroscopy to confirm reduction and place interlocking screws, which is associated with a learning curve [9, 22–24, 26–29, 31]. Fluoroscopy time can decrease by almost 80% and operative time by over 40% with experience [32–34]. Utilizing interlocking screw guides also decreases fluoroscopy use [27].

While the difference in operative blood loss may be of minimal clinical importance, the lack of soft tissue dissection during IMN is a notable advantage to wound healing [26, 27, 35]. Periosteal stripping during ORIF can delay the normalization of blood flow at the fracture site and impair fracture healing [36]. In addition, soft tissue damage can lead to increased swelling, pain, and wound complications [37, 38]. ORIF had a mean incision size 330% larger than IMN and a 250% larger periosteal stripping area, which could be a possible reason for increased time-to-union in some patients with ORIF [22, 24–27]. In addition, biomechanical analysis of IMN and ORIF for isolated ulna fractures has shown that IMN has lower yet sufficient bending and torsional stiffness but greater axial stiffness than plate fixation [39]. In combination with less periosteal stripping, maintaining the fracture hematoma with intramedullary stabilization could improve healing, especially in comminuted fractures [39, 40].

### Complications and implant removal

**Fig. 25:**
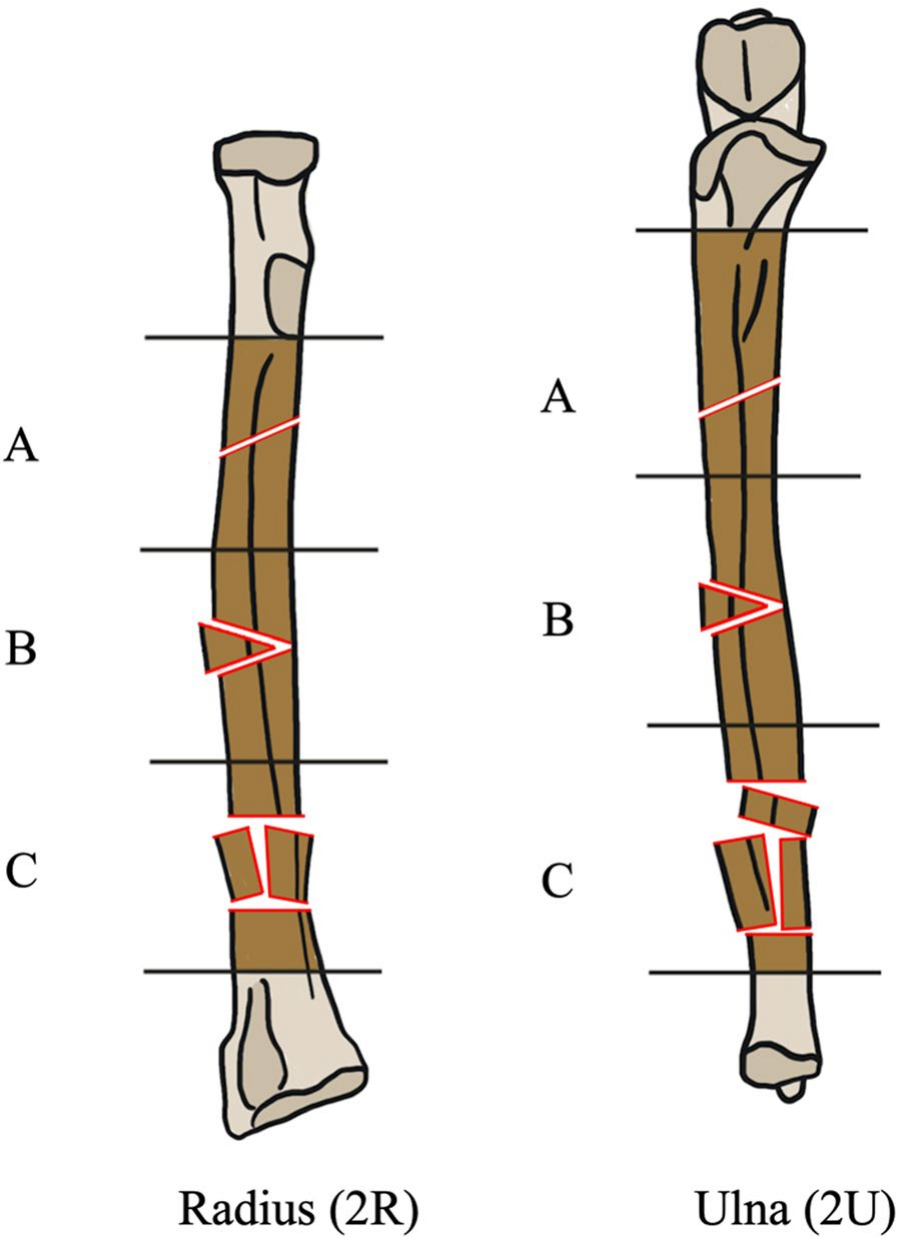
AO/OTA classification of radius and ulna diaphyseal fractures (Artwork by Lauren Domingue)

IMN had lower rates of overall complications, SSI, and implant removal than ORIF. The most common complication reported overall was SSI. One deep infection was reported in a patient treated with ORIF [24]. The mean infection rate overall for IMN groups in this study (1.8%) was similar to previous ORIF literature (2–3.5%) [30, 41, 42]. However, the mean infection rate for ORIF groups in this study (9.1%) was higher [9, 24, 26–29]. While the percentage of open fractures in ORIF and IMN was similar to the previous ORIF literature, there was a higher percentage of AO/OTA type C fractures [Type 2R/U2A: simple fracture, Type 2R/U2B: wedge fracture, Type 2R/U2C: multifragmentary (i.e. comminuted) fracture] (Fig. 25) in ORIF than in the IMN group and may be partly responsible for the increased infection rate in ORIF [9, 24, 26–29]. IMN may be particularly useful for AO/OTA type C fractures to lower the risk of infection through decreased soft tissue exposure and shorter procedure length.

Injury to the EPL tendon is a potential complication of radial IMN implants that use an entry point around Lister’s tubercle [43, 44]. This study reported one case of late EPL rupture due to wear from the nail tip [27]. Identifying and protecting the EPL tendon during IMN entry and seating the head of the radial IMN flush with the cortex can reduce this complication [44].

Three cases of posterior interosseus nerve (PIN) injury occurred in patients treated with a radial nail requiring proximal locking screws, which can end up in close proximity to the PIN [9, 22]. This risk can be avoided with radial IMN designs that do not require proximal interlocking screws and provide proximal rotational stability by locking into the metaphysis via a blade tip. One case of nerve palsy of the superficial branch of the radial nerve was reported due to damage when placing distal interlocking screws in radial IMNs [24].

IMN hardware was significantly less likely to be removed than ORIF in this meta-analysis. A key advantage of an IMN over ORIF is decreased implant irritation necessitating hardware removal [23]. Plate removal increases the risk of refracture and historically occurs in up to 22% of cases within the first year after removal [6, 45–48]. There were no refractures after IMN removal in the current analysis, but there was one incidence of refracture seven months after plate removal [9]. Plates are stress-shielding constructs, but intramedullary nails are stress-sharing, forming a callus that increases the diameter and strength of bone at the fracture site compared to the pre-fracture state. Also, IMN removal does not leave residual bicortical screw holes near the fracture site, which may increase the risk of refracturing [12]. Additionally, IMN removal does not require postoperative immobilization, while plate removal may be accompanied by immobilization [49].

### Radiographic outcomes

The meta-analysis showed that IMN trended towards faster time-to-union and similar nonunion rates than ORIF. The time-to-union of IMN in these studies was similar to or less than those reported in larger population ORIF studies [30, 42]. Faster time-to-union could be explained by decreased periosteum disruption and earlier mobilization with IMN [9, 22, 25, 27]. In contrast, previous series on non-locked forearm IMN models had high rates of nonunion and required prolonged immobilization due to rotational instability [1, 12]. In all studies included in this review where IMN was not immobilized, time-to-union was equal to or less than ORIF [24, 25, 27–29]. Thus, early mobilization should be recommended after interlocking IMN in most cases [22, 24–29].

### Functional outcomes

This meta-analysis demonstrated improved Grace–Eversmann scores with IMN and similar DASH scores, forearm ROM, and grip strength between IMN and ORIF [9, 24, 27–29]. In three studies, DASH scores were lower in ORIF, but none reached the minimal clinical difference of 11 points [9, 26, 27]. Overall, DASH scores were consistent with previous ORIF literature [10, 37, 49, 50]. The Grace–Eversmann rating system is a joint-specific measure of pronosupination and union (Table 6), which may be a more appropriate assessment for forearm fractures than DASH scores [51]. Pavone et al. [25] reported significantly lower (better) DASH scores at one and three months, significantly less physical therapy usage, and faster return to work or sport in IMN for isolated ulna fractures. These results could be partly due to quicker time-to-union and earlier mobilization with IMN [25].

**Table 6:** Grace–Eversmann criteria

Rating	Rotation arc to contralateral side (%)	Union Status
Excellent	≥ 90	Union
Good	80–89	Union
Acceptable	60–79	Union
Poor	< 60	Nonunion

Forearm ROM is affected by reduction accuracy, restoration of the radial bow, and mobilization. Restoring the forearm bones to within 10 degrees of normal angulation in all planes has been shown to avoid any significant negative impact on patient function due to alignment [3]. Anatomic reduction and restoration of the radial bow have been previous concerns with IMN since closed methods are typically utilized for fracture reduction [12]. Poor reduction, therefore, has implications for limiting forearm ROM. Studies show that losses of up to 2 mm of the radial bow magnitude do not affect functional outcomes [52, 53]. In addition, exact restoration of the radial bow with ORIF can still lead to a limited range of motion due to soft tissue fibrosis, scarring, adhesions, and delayed mobilization [54]. Early ROM is a stronger determinant of forearm function than radial bow restoration [3, 32, 55, 56]. There is mixed evidence of the effect of radial bow changes on grip strength [14, 32, 56].

**Fig. 26:**
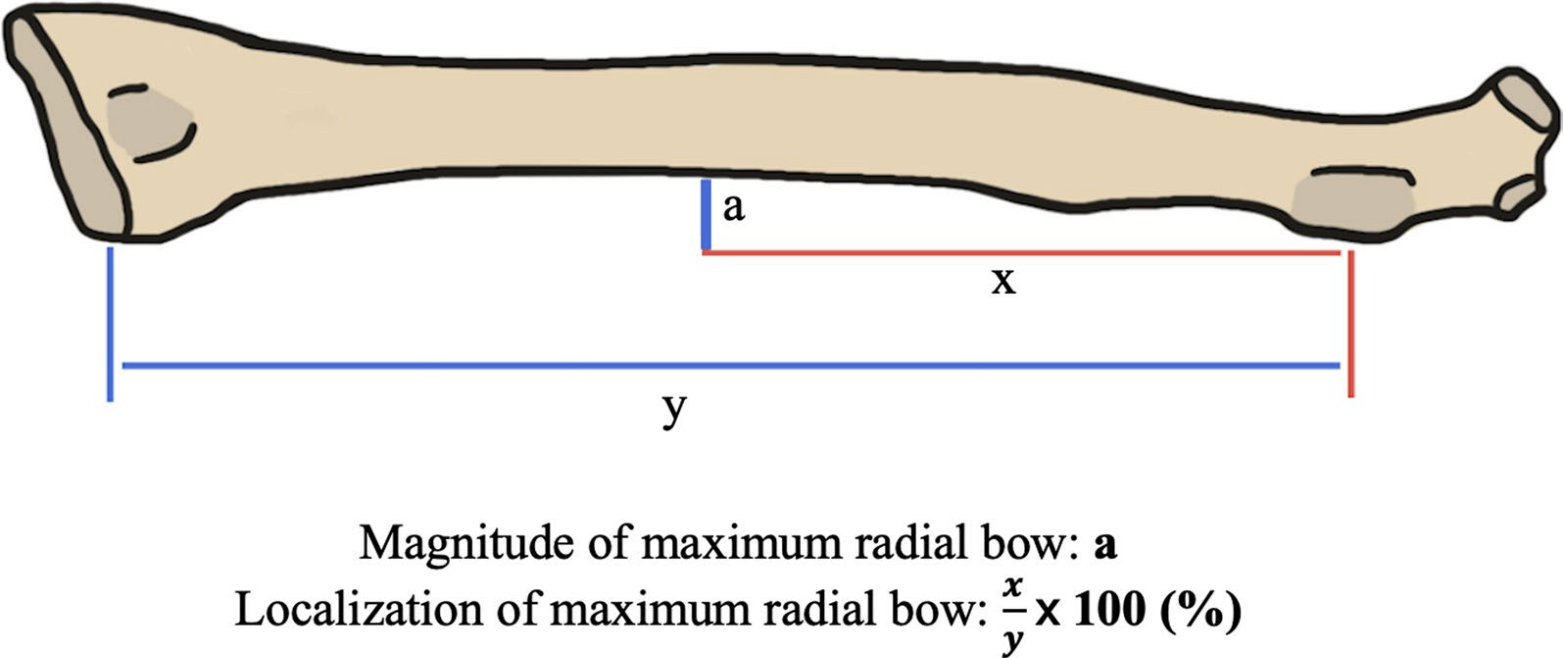
Magnitude and localization of maximum radial bow measurement example and equations (Artwork by Lauren Domingue)

Two studies in this review reported the magnitude of the radial bow [9, 27]. Lee et al. [9] compared the injured arm to the contralateral arm to analyze the restoration of the radial bow. The ORIF group had significantly improved radial bow restoration than the IMN group (95.0 ± 4.7 vs. 90.0 ± 3.5; *P* = 0.043) [9]. There was a significantly lower difference in the mean ratio of radial bow localization (i.e. apex location of maximal bow in the radius and ulna expressed as a ratio to each other) (Fig. 26) of the injured side to the contralateral side in the ORIF group compared to the IMN group (1.0 ± 1.4 vs. 1.1 ± 3.6; *P* = 0.017) [9].

In Köse et al. [27] and Lee et al. [9], changes in the radial bow had no relationship to changes in pronosupination, which is consistent with previous IMN studies [9, 27, 32, 56]. Thus, while IMN has decreased accuracy in restoring radial bow, changes in radial bow do not necessarily translate into clinical differences in outcome (Fig. 26).

### Limitations

There were several limitations in this meta-analysis. First, only English-available articles were considered. Second, regardless of a comprehensive search, only 2 RCTs were found, with a risk of bias because of unblinded surgeons, patients, and staff. Third, the inclusion of non-randomized studies decreases the level of evidence of findings, and the studies individually involve smaller groups of patients in select countries. However, this is why a systematic review and meta-analysis were performed to pool results from several smaller studies to help better understand treatment outcomes. Fourth, the weight of the RCTs in the meta-analysis was based on the number of patients and not on quality or bias compared to the non-randomized trials. While the results of the RCTs should carry more weight in a meta-analysis, there is currently no accepted method of weighing studies based on quality, and the fact that study quality is subjective could lead to further bias in the results. Fifth, heterogeneity in patient populations concerning IMN brand and fracture classification, open fracture makeup, and rehabilitation protocols between IMN and ORIF can be confounding variables. Further studies should strive to analyze results with standardized variables to determine the optimal situations for IMN use.

### Applications for future research

More RCTs could be performed to compare outcomes in more homogenous patient populations. This will allow information to help better define indications for IMN use. Given that IMN trends towards faster recovery and return to work, fewer complications, and potentially less physical therapy, economic studies need to be performed to understand the overall cost–benefit analysis.

## Conclusions

The findings of this meta-analysis were based on the highest quality studies currently available comparing interlocked IMN to ORIF for forearm diaphyseal fractures. There is a trend towards faster time-to-union with IMN and similar nonunion rates between IMN and ORIF. IMN had statistically significant but not clinically significant lower operative times. IMN demonstrated lower complication and SSI rates and improved Grace–Eversmann scores. Other functional outcomes were similar between IMN and ORIF. ORIF has been considered the gold standard due to anatomic reduction, fixation, and restoration of the radial bow. Based on these findings, interlocking IMN fixation of forearm diaphyseal fractures has similar or improved outcomes to ORIF and should be considered a safe and effective treatment option. Further, higher-quality studies should be performed to compare outcomes between these two treatment modalities for forearm diaphyseal fractures in adults.

## Supplementary Information

Supplementary file 1.

Supplementary file 2.

Supplementary file 3.

Supplementary file 4.

## Data Availability

All data generated or analyzed during this study are included in this published article [and its supplementary information files].
