# Supplementary material for: Intramedullary nail fixation versus open reduction and internal fixation for treatment of adult diaphyseal forearm fractures: a systematic review and meta-analysis
Source: J Orthop Surg Res. 2024 Nov 4;19:719. doi: 10.1186/s13018-024-05158-0 (PMC11533272; doi:10.1186/s13018-024-05158-0)
Supplement: Supplementary file 1 [file 13018_2024_5158_MOESM1_ESM.docx]

**Appendix A: Risk of Bias Analysis**

|  | Lee et al 2014 | Zhang et al 2016 |  |  |
| --- | --- | --- | --- | --- |
| Randomization process | 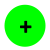 | 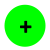 | 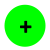 | Low risk |
| Deviations from the intended interventions | 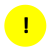 | 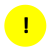 | 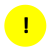 | Some concerns |
| Missing outcome data | 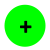 | 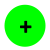 | 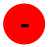 | High risk |
| Measurement of the outcome | 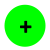 | 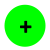 |  |  |
| Selection of the reported result | 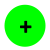 | 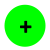 |  |  |
|  |  |  |  |  |
| **Overall** | **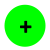** | **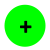** |  |  |
|  |  |  |  |  |

***Appendix A Figure 1****: Rob 2.0. Cochrane Risk of Bias: (A) Graph, (B) Summary*

***Appendix A Table 1: The risk of bias in nonrandomized studies of interventions (ROBINS-I) of non-randomized trials***

| Study | Domains |  |  |  |  |  |  |  |
| --- | --- | --- | --- | --- | --- | --- | --- | --- |
|  | Pre-intervention | | Intervention | Post-intervention | | | |  |
|  | Bias due to confounding | Bias in selection of participants into the study | Bias in classification on interventions | Bias due to deviations from intended interventions | Bias due to missing data | Bias in the measurement of the outcome | Bias in selection of reported results | Overall Risk of Bias |
| Kibar & Kurtulmuş^25^ | Moderate: Surgeon determined intervention: less A type and more B type in IMN | Low | Low | Low | Low | Moderate: Outcome measure minimally influenced by knowledge of the intervention | Low | Moderate |
| Kibar & Kurtulmuş^26^ | Moderate: Surgeon determined intervention; more open fractures in IMN | Low | Low | Low | Low | Moderate: Outcome measure minimally influenced by knowledge of the intervention | Low | Moderate |
| Köse et al^24^ | Moderate: Surgeon determined intervention; more open fractures in IMN | Serious: inclusion and exclusion definition did not control for type of fracture | Low | Low | Low | Moderate: Outcome measure minimally influenced by knowledge of the intervention | Serious: Data not separated for type of fracture | Serious |
| Ozkaya et al^23^ | Serious: Surgeon determined intervention; AO classification not controlled for | Serious: No mention of inclusion and exclusion criteria | Low | Serious: Post-operative immobilization was determined based on surgeon determination of "rigidity" of fixation in IMN | Moderate, did not report AO classification breakdown in IMN and ORIF | Moderate: Outcome measure minimally influenced by knowledge of the intervention | Low | Serious |
| Pavone et al^22^ | Moderate: Surgeon determined intervention | Low | Low | Low | Low* | Moderate: Outcome measure minimally influenced by knowledge of the intervention | Low | Moderate |
| Polat & Toy^21^ | Moderate: Surgeon determined intervention | Low | Low | Low | Low | Moderate: Outcome measure minimally influenced by knowledge of the intervention | Low | Moderate |
| Sisman & Polat^20^ | Moderate: Surgeon determined intervention | Low | Low | Low | Serious: did not report time to union, only if achieved radiographic healing | Moderate: Outcome measure minimally influenced by knowledge of the intervention | Low | Serious |
|  |  |  |  |  |  |  |  |  |
| *Although AO classification not reported, the definition of fracture inclusion was equivalent to type A fractures. | | | | | | |  |  |
