# Supplementary material for: Intramedullary nail fixation versus open reduction and internal fixation for treatment of adult diaphyseal forearm fractures: a systematic review and meta-analysis
Source: J Orthop Surg Res. 2024 Nov 4;19:719. doi: 10.1186/s13018-024-05158-0 (PMC11533272; doi:10.1186/s13018-024-05158-0)
Supplement: Supplementary file 2 [file 13018_2024_5158_MOESM2_ESM.docx]

***Table 1: Studies that evaluated each outcome for all fractures, both-bone forearm fractures, and isolated ulna fractures***

| **Outcome** | **All Studies** | **BBFF Studies** | **Isolated Ulna Studies** |
| --- | --- | --- | --- |
| **Operative Time** | Kibar & Kurtulmuş^28^ Kibar & Kurtulmuş^29^ Köse et al^27^ Lee et al^9^ Ozkaya et al^26^ Sisman & Polat^23^ Polat & Toy^24^ Zhang et al^22^ | Lee et al^9^ Ozkaya et al^26^ Polat & Toy^24^ Zhang et al^22^ | Kibar & Kurtulmuş^29^ Sisman & Polat^23^ |
| **Complications** | | | |
| **Complications** | Kibar & Kurtulmuş^28^ Kibar & Kurtulmuş^29^ Köse et al^27^ Lee et al^9^ Ozkaya et al^26^ Pavone et al^25^ Polat & Toy^24^ Sisman & Polat^23^ Zhang et al^22^ | Lee et al^9^ Ozkaya et al^26^ Polat & Toy^24^ Zhang et al^22^ | Kibar & Kurtulmuş^29^ Pavone et al^25^ Sisman & Polat^23^ |
| **Surgical Site Infection** | Kibar & Kurtulmuş^28^ Kibar & Kurtulmuş^29^ Köse et al^27^ Lee et al^9^ Ozkaya et al^26^ Pavone et al^25^ Polat & Toy^24^  Sisman & Polat^23^ Zhang et al^22^ | Lee et al^9^ Ozkaya et al^26^ Polat & Toy^24^ Zhang et al^22^ | Kibar & Kurtulmuş^29^ Pavone et al^25^ Sisman & Polat^23^ |
| **Implant Removal** | Kibar & Kurtulmuş^28^ Kibar & Kurtulmuş^29^ Köse et al^27^ Lee et al^9^  Ozkaya et al^26^ Pavone et al^25^ Polat & Toy^24^ Sisman & Polat^23^ | Lee et al^9^ Ozkaya et al^26^ Polat & Toy^24^ | Kibar & Kurtulmuş^29^ Pavone et al^25^ Sisman & Polat^23^ |
| **Radiographic Outcomes** | | | |
| **Time-to-Union** | Kibar & Kurtulmuş^28^ Kibar & Kurtulmuş^29^  Köse et al^27^  Lee et al^9^ Ozkaya et al^26^ | Lee et al^9^ Ozkaya et al^26^ | Kibar & Kurtulmuş^29^ |
| **Nonunion Rate** | Kibar & Kurtulmuş^28^  Kibar & Kurtulmuş^29^ Köse et al^27^ Lee et al^9^ Ozkaya et al^26^ Pavone et al^25^ Polat & Toy^24^ Sisman & Polat^23^ Zhang et al^22^ | Lee et al^9^ Ozkaya et al^26^ Polat & Toy^24^ Zhang et al^22^ | Kibar & Kurtulmuş^29^ Pavone et al^25^ Sisman & Polat^23^ |
| **Functional Outcomes** | | | |
| **DASH Score** | Kibar & Kurtulmuş^28^ Kibar & Kurtulmuş^29^ Köse et al^27^ Lee et al^9^ Ozkaya et al^26^ Pavone et al^25^ Polat & Toy^24^ Sisman & Polat^23^ | Lee et al^9^ Ozkaya et al^26^ Polat & Toy^24^ | Kibar & Kurtulmuş^29^ Pavone et al^25^ Sisman & Polat^23^ |
| **Excellent or Good Grace-Eversmann Score** | Kibar & Kurtulmuş^28^ Kibar & Kurtulmuş^29^ Köse et al^27^  Lee et al^9^ Ozkaya et al^26^  Polat & Toy^24^ Sisman & Polat^23^ Zhang et al^22^ | Lee et al^9^ Ozkaya et al^26^ Polat & Toy^24^ Zhang et al^22^ | Kibar & Kurtulmuş^29^ Sisman & Polat^23^ |
| **Pronosupination ROM** | Kibar & Kurtulmuş^28^ Kibar & Kurtulmuş^29^ Köse et al^27^ Lee et al^9^ Polat & Toy^24^ | Lee et al^9^ Polat & Toy^24^ | Kibar & Kurtulmuş^29^ |
| **Grip Strength** | Kibar & Kurtulmuş^28^ Kibar & Kurtulmuş^29^ Köse et al^27^ | N/A | Kibar & Kurtulmuş^29^ |

*(BBFF, Both-bone forearm fracture; IMN, Intramedullary Nail; ORIF, Open Reduction and Internal Fixation)*
