# Supplementary material for: Intramedullary nail fixation versus open reduction and internal fixation for treatment of adult diaphyseal forearm fractures: a systematic review and meta-analysis
Source: J Orthop Surg Res. 2024 Nov 4;19:719. doi: 10.1186/s13018-024-05158-0 (PMC11533272; doi:10.1186/s13018-024-05158-0)
Supplement: Supplementary file 3 [file 13018_2024_5158_MOESM3_ESM.docx]

**Appendix C**

To identify influential studies and assess their impact, a sensitivity analysis was performed using the leave-one-out method. The resulting Baujat plots are shown.

**Operation Time**

Zhang et al. (2016) and Ozkaya et al. (2009) are furthest to the right, indicating these are relatively large sources of heterogeneity. Furthermore, Ozkaya et al. (2009) is furthest along the vertical axis too, indicating its has the largest influence on the overall effect size. To assess their relative impact, the leave-one-out effect size analyses are shown below. Note that both studies identified as potentially being influential result in a larger effect size when excluded. If anything, these studies have a moderating effect on the analysis.

***Table 1: Leave-One-Out Sensitivity Analysis***

| Study Excluded | SMD | 95% CI Lower | 95% CI Upper |
| --- | --- | --- | --- |
| Ozkaya (2009)*^,^** | -2.028 | -2.791 | -1.264 |
| Lee (2014) | -1.735 | -2.629 | -0.840 |
| Zhang (2016)* | -1.559 | -2.254 | -0.864 |
| Köse (2017) | -1.931 | -2.818 | -1.044 |
| Kibar (2019) | -1.900 | -2.802 | -0.998 |
| Kibar (2020) | -1.793 | -2.705 | -0.880 |
| Polat (2022) | -1.937 | -2.815 | -1.059 |
| Şişman (2023) | -1.634 | -2.440 | -0.828 |

*(*Large source of heterogeneity; **Large influence on the overall effect size)*

***Figure 1: Baujat Plot of Operative Time Sensitivity Analysis***

**Complications**

The leave-one-out method identifies Zhang et al (2016) and as most influential on the effect size, while Şişman et al (2023) is the most influential on study heterogeneity. The leave-one-out OR analysis in the table indicates that excluding Zhang results in a stronger effect (risk reduction) according to the pooled OR. Excluding Şişman et al (2023) results in a weaker effect and the effect is no longer significant at α= 0.05 as the resulting 95% confidence interval spans 1, an OR value indicating no effect.

***Table 2: Complications Leave-One-Out Sensitivity Analysis***

| Study Excluded | OR | 95% CI Lower | 95% CI Upper |
| --- | --- | --- | --- |
| Ozkaya (2009) | 0.456 | 0.243 | 0.859 |
| Lee (2014) | 0.418 | 0.219 | 0.796 |
| Zhang (2016)** | 0.402 | 0.204 | 0.795 |
| Köse (2017) | 0.467 | 0.248 | 0.880 |
| Kibar (2019) | 0.531 | 0.286 | 0.987 |
| Kibar (2020) | 0.547 | 0.293 | 1.019 |
| Pavone (2021) | 0.476 | 0.259 | 0.875 |
| Polat (2022) | 0.433 | 0.223 | 0.841 |
| Şişman (2023)* | 0.566 | 0.302 | 1.060 |

*(*Large source of heterogeneity; **Large influence on the overall effect size)*

***Figure 2: Baujat Plot of Complications Sensitivity Analysis***

**Surgical Site Infection**

The leave-one-out method identifies Polat et al (2022) as being the most influential, both in terms of heterogeneity and impact on the effect size (see figure). The leave-one-out OR analysis indicates a much stronger pooled effect when this study is excluded

***Table 3: Surgical Site Infection Leave-One-Out Sensitivity Analysis***

| Study Excluded | OR | 95% CI Lower | 95% CI Upper |
| --- | --- | --- | --- |
| Ozkaya (2009) | 0.314 | 0.13 | 0.758 |
| Lee (2014) | 0.303 | 0.126 | 0.728 |
| Zhang (2016) | 0.337 | 0.139 | 0.818 |
| Köse (2017) | 0.290 | 0.117 | 0.716 |
| Kibar (2019) | 0.313 | 0.130 | 0.756 |
| Kibar (2020) | 0.323 | 0.134 | 0.782 |
| Pavone (2021) | 0.293 | 0.122 | 0.704 |
| Polat (2022)*^,^** | 0.210 | 0.074 | 0.593 |
| Şişman (2023) | 0.340 | 0.140 | 0.828 |

*(*Large source of heterogeneity; **Large influence on the overall effect size)*

***Figure 3: Baujat Plot of SSI Sensitivity Analysis***

**Implant Removal**

The leave-one-out method identifies Köse et al (2017) as being the influential both in terms of heterogeneity and impact on the effect size, and Şişman et al (2023) is influential in terms of study heterogeneity (see figure). The leave-one-out OR analysis indicates a much stronger pooled effect when Köse is excluded, and a weaker effect when Şişman is excluded, while still being statistically significant.

***Table 4: Implant Removal Leave-One-Out Sensitivity Analysis***

| Study Excluded | OR | 95% CI Lower | 95% CI Upper |
| --- | --- | --- | --- |
| Ozkaya (2009) | 0.345 | 0.148 | 0.803 |
| Lee (2014) | 0.294 | 0.135 | 0.639 |
| Köse (2017)*^,^** | 0.239 | 0.102 | 0.558 |
| Kibar (2019) | 0.323 | 0.156 | 0.670 |
| Kibar (2020) | 0.328 | 0.158 | 0.682 |
| Polat (2022) | 0.346 | 0.166 | 0.723 |
| Şişman (2023)* | 0.411 | 0.193 | 0.878 |

*(*Large source of heterogeneity; **Large influence on the overall effect size)*

***Figure 4: Baujat Plot of Implant Removal Sensitivity Analysis***

**Time-To-Union**

The leave-one-out method identifies Lee et al (2014) as the most influential both with regards to study heterogeneity and impact on effect size. Excluding this results in a stronger effect, and it also becomes statistically significant (in favor of IMN having shorter time-to-union) α= 0.05.

***Table 5: Time-To-Union Leave-One-Out Sensitivity Analysis***

| Study Excluded | SMD | 95% CI Lower | 95% CI Upper |
| --- | --- | --- | --- |
| Ozkaya (2009) | -0.178 | -0.986 | 0.630 |
| Lee (2014)*^,^** | -0.901 | -1.673 | -0.129 |
| Köse (2017) | -0.456 | -1.681 | 0.769 |
| Kibar (2019) | -0.507 | -1.744 | 0.729 |
| Kibar (2020) | -0.137 | -0.952 | 0.678 |

*(*Large source of heterogeneity; **Large influence on the overall effect size)*

***Figure 5: Baujat Plot of Time-To-Union Sensitivity Analysis***

**Non-Union Rate**

The leave-one-out method identifies Lee et al (2014) as being influential both in terms of heterogeneity and impact on the effect size (see figure). The leave-one-out OR analysis indicates a much stronger pooled effect when Lee is excluded, but the result remains inconclusive due to a lack of statistical significance.

***Table 6: Non-Union Leave-One-Out Sensitivity Analysis***

| Study Excluded | OR | 95% CI Lower | 95% CI Upper |
| --- | --- | --- | --- |
| Lee (2014)*^,^** | 0.317 | 0.063 | 1.604 |
| Köse (2017) | 0.585 | 0.136 | 2.517 |
| Kibar (2019) | 0.554 | 0.130 | 2.367 |
| Kibar (2020) | 0.596 | 0.139 | 2.561 |
| Polat (2022) | 0.546 | 0.128 | 2.327 |

*(*Large source of heterogeneity; **Large influence on the overall effect size)*

***Figure 6: Baujat Plot of Non-Union Sensitivity Analysis***

**DASH Scores**

The leave-one-out method Şişman et al (2023) as the most influential both with regards to study heterogeneity and impact on effect size. Excluding Şişman et al (2023) results in a stronger effect, but it is still not detected to statistically significant at $\alpha=$ 0.05.

***Table 7: DASH Leave-One-Out Sensitivity Analysis***

| Study Excluded | SMD | 95% CI Lower | 95% CI Upper |
| --- | --- | --- | --- |
| Ozkaya (2009) | -0.269 | -1.042 | 0.504 |
| Lee (2014) | -0.321 | -1.043 | 0.402 |
| Zhang (2016) | -0.230 | -1.034 | 0.573 |
| Köse (2017) | -0.165 | -0.982 | 0.653 |
| Kibar (2019) | -0.137 | -0.952 | 0.678 |
| Kibar (2020) | -0.143 | -0.950 | 0.663 |
| Pavone (2021) | -0.142 | -0.957 | 0.674 |
| Polat (2022) | 0.177 | -0.205 | 0.560 |
| Şişman (2023)*^,^** | -0.269 | -1.042 | 0.504 |

*(*Large source of heterogeneity; **Large influence on the overall effect size)*

***Figure 7: Baujat Plot of DASH Sensitivity Analysis***

**Grace-Eversmann (Excellent & Good Ratings)**

The leave-one-out method identifies Zhang et al (2016) and Lee et al (2014) as possibly influential. However, leaving out these studies has minimal impact on the resulting pooled OR and does not change the statistical significance of the results.

***Table 8:GE Score Leave-One-Out Sensitivity Analysis***

| Study Excluded | OR | 95% CI Lower | 95% CI Upper |
| --- | --- | --- | --- |
| Ozkaya (2009) | 2.243 | 1.056 | 4.762 |
| Lee (2014) | 2.537 | 1.207 | 5.332 |
| Zhang (2016) | 2.673 | 1.160 | 6.161 |
| Köse (2017) | 2.051 | 0.999 | 4.210 |
| Kibar (2019) | 2.047 | 0.981 | 4.268 |
| Kibar (2020) | 2.119 | 0.997 | 4.505 |
| Polat (2022) | 2.010 | 0.976 | 4.138 |
| Şişman (2023) | 2.149 | 1.053 | 4.384 |

***Figure 8: Baujat Plot of GE Score Sensitivity Analysis***
