# Supplementary material for: Intramedullary nail fixation versus open reduction and internal fixation for treatment of adult diaphyseal forearm fractures: a systematic review and meta-analysis
Source: J Orthop Surg Res. 2024 Nov 4;19:719. doi: 10.1186/s13018-024-05158-0 (PMC11533272; doi:10.1186/s13018-024-05158-0)
Supplement: Supplementary file 4 [file 13018_2024_5158_MOESM4_ESM.docx]

**Appendix D: Publication Bias Assessment**

**Operative Time**

The meta-analysis of operative time was based on eight studies and found a statistically significant SMD of -1.813 [-2.602; -1.024]. The trim-and-fill method added two hypothetical studies to adjust for potential publication bias, resulted in an SMD of -1.302 [-2.245; -0.358], with the effect remaining statistically significant. Egger’s test for funnel plot asymmetry resulted in a t-value of -2.733 with p-value 0.034, suggesting some evidence of publication bias that may have influenced the observed heterogeneity.

***Figure 1: Funnel plots used describe heterogeneity for operative time***

**Complications**

The meta-analysis of complications was based on nine studies and found a significant OR of 0.476 [0.262; 0.867]. The trim-and-fill method added three hypothetical studies to adjust for potential publication bias, resulted in an OR of 0.813 [0.442; 1.495], with the adjusted effect no longer being statistically significant. Egger’s test showed a t-value of -3.386 with p-value 0.012, suggesting some evidence of publication bias that may have influenced the observed heterogeneity.

***Figure 2: Funnel plots used describe heterogeneity for complications***

**Surgical Site Infections**

The meta-analysis of SSI was based on nine studies and found a non-significant OR of 0.302 [0.130; 0.705]. The trim-and-fill method added five hypothetical studies to adjust for potential publication bias, resulted in an estimated OR of 0.597 [0.283; 1.257] that was no longer statistically significant. Egger’s test showed a t-value of -3.931 with p-value 0.006, suggesting some evidence of publication bias that may have influenced the observed heterogeneity.

***Figure 3: Funnel plots used describe heterogeneity for surgical site infections***

**Implant Removal**

The meta-analysis of implant removal was based on seven studies and found a significant OR of 0.325 [0.159; 0.662]. The trim-and-fill method added two hypothetical studies to adjust for potential publication bias, resulted in an estimated OR of 0.460 [0.226; 0.935], with the effect remaining statistically significant. Egger’s test showed a t-value of -1.333 with p-value 0.240, suggesting that publication bias is unlikely to account for the observed heterogeneity.

***Figure 4: Funnel plots used describe heterogeneity for implant removal***

**Time-To-Union**

The meta-analysis of time-to-union, based on five studies, indicates a non-significant SMD of -0.537 [-1.492; 0.418]. The trim-and-fill method, which added one hypothetical study to adjust for potential publication bias, resulted in an SMD of -0.193 [-1.238; 0.853], confirming the non-significant effect. Egger’s test for funnel plot asymmetry showed a t-value of -0.693 and a p-value of 0.538, suggesting that publication bias is unlikely to account for the observed heterogeneity.

***Figure 5: Funnel plots used describe heterogeneity for time-to-union***

**Non-Union Rates**

The meta-analysis of non-union rates was based on five studies and found an OR of 0.512 [0.137; 1.915]. The trim-and-fill method added one hypothetical study to adjust for potential publication bias, resulted in an estimated OR of 0.576 [0.153; 2.163], with the effect remaining non-significant. Egger’s test showed a t-value of -0.432 with p-value 0.695, suggesting that publication bias is unlikely to account for the observed heterogeneity.

***Figure 6: Funnel plots used describe heterogeneity for non-union rates***

**DASH Scores**

The meta-analysis of DASH scores was based on eight studies and found a non-significant SMD of -0.154 [-0.858; 0.550]. The trim-and-fill method did not add any hypothetical studies and thus no adjustment was incorporated. Egger’s test showed a t-value of -1.524 with p-value 0.178, suggesting that publication bias is unlikely to account for the observed heterogeneity.

***Figure 7: Funnel plots used describe heterogeneity for DASH scores***

**Grace-Eversmann Scores**

The meta-analysis of GE scores was based on eight studies and found an OR of 2.206 [1.100; 4.422]. The trim-and-fill method added two hypothetical study to adjust for potential publication bias, resulted in an estimated OR of 1.810 [0.906; 3.616], with the adjusted effect becoming non-significant. However, Egger’s test showed a t-value of 1.524 with p-value 0.178, suggesting that publication bias is unlikely to account for the observed heterogeneity and the original finding is plausible.

***Figure 8: Funnel plots used describe heterogeneity for Grace-Eversmann scores***
